# White Adipose Tissue Contributes to Cardiac Homeostasis and Adaptation to Cardiometabolic Stress in a Sex- and Depot-Specific Manner

**DOI:** 10.3390/biom16091364

**Published:** 2026-09-19

**Authors:** Sara-Ève Thibodeau, Nicolas Le Bos, Élisabeth Walsh-Wilkinson, Emylie-Ann Labbé, Audrey Morin-Grandmont, Emmy Trahan, Mathias Zakrzewski, Jacques Couet

**Affiliations:** 1 Département de Médecine, Faculté de Médecine, Université Laval, Québec City, QC G1V 0A6, Canada; sara-eve.thibodeau@criucpq.ulaval.ca (S.-È.T.); nicolas.le-bos.1@ulaval.ca (N.L.B.); elisabeth.walsh-wilkinson.1@ulaval.ca (É.W.-W.); emylie-ann.labbe.1@ulaval.ca (E.-A.L.); audrey.morin-grandmont@criucpq.ulaval.ca (A.M.-G.); emmy.trahan.1@ulaval.ca (E.T.); mathias.zakrzewski.1@ulaval.ca (M.Z.); 2 Groupe de Recherche sur les Valvulopathies, Centre de Recherche de l’Institut Universitaire de Cardiologie et de Pneumologie de Québec, Université Laval, Québec City, QC G1V 4G5, Canada

**Keywords:** heart failure, fat depots, white adipose tissue, lipectomy, cardiac hypertrophy, mouse, sex differences, HFpEF

## Abstract

Background. White adipose tissue (WAT) is increasingly recognized as an endocrine organ involved in cardiovascular physiology. However, the specific contributions of distinct adipose depots to cardiac homeostasis and adaptation to pathological stress remain poorly understood. We investigated the consequences of visceral (VAT) or subcutaneous (SAT) lipectomy (Lpx) on cardiac phenotype in male and female C57BL/6J mice and evaluated its impact on the response to a stress-inducing heart failure with preserved ejection fraction-like phenotype. Methods. VAT or SAT depot removal was performed in young adult mice. Ten weeks later, we assessed cardiac morphology, function, circulating adipokines, and left ventricular (LV) gene expression. We then exposed separate cohorts to angiotensin II infusion combined with a high-fat diet. We also conducted experiments in ovariectomized (Ovx) females to evaluate the contribution of ovarian hormones. Results. Lpx induced marked depot- and sex-specific effects on body composition, cardiac morphology, and echocardiographic parameters. Changes in LV mass, atrial size, and diastolic indices were observed despite the absence of overt pathology. These alterations were accompanied by substantial modulation of genes involved in cardiac hypertrophy, calcium handling, extracellular matrix remodelling, and energy metabolism. VAT and SAT removal also differentially affected circulating adiponectin, leptin, and MCP-1 levels. Lpx modified the cardiac hypertrophic response to cardiometabolic stress in a sex- and depot-dependent manner. Ovx altered several of these responses and was associated with evidence of increased pulmonary congestion in VAT-lipectomized females. Conclusions. These findings show that WAT contributes to maintaining cardiac homeostasis and influences adaptation to pathological stress through mechanisms highly dependent on adipose depot location, biological sex, and ovarian hormone status.

## 1. Introduction

Heart failure with preserved ejection fraction (HFpEF) is a multifactorial syndrome characterized by cardiac hypertrophy, concentric remodelling, left atrial enlargement and impaired diastolic function despite preserved systolic performance [1]. In addition to ageing and hypertension, obesity and metabolic dysfunction are major risk factors for HFpEF, particularly in women [2]. Increasing evidence suggests that HFpEF should not be viewed exclusively as a cardiac disease but rather as a systemic disorder involving complex interactions between the heart and peripheral organs.

White adipose tissue (WAT) is now recognized as an active endocrine organ that regulates whole-body physiology by secreting adipokines, cytokines, lipids and extracellular vesicle-associated molecules, including microRNAs [3,4]. Through these mechanisms, adipose tissue helps regulate inflammation, energy metabolism, vascular physiology and tissue remodelling. Excess adiposity is generally associated with adverse cardiovascular outcomes; however, the physiological contribution of normal adipose depots to cardiac homeostasis remains incompletely understood [5].

An additional layer of complexity arises from adipose tissue heterogeneity. Visceral and subcutaneous depots differ markedly in developmental origin, cellular composition, endocrine profile and metabolic activity [6]. Clinical and experimental studies suggest that these depots may exert distinct cardiovascular effects [7]. Furthermore, important sex differences exist in adipose tissue distribution and function, indicating that adipose–cardiac communication may be strongly influenced by biological sex and sex hormones [8].

Most available studies have focused on the consequences of obesity or adipose tissue expansion [3,5]. In contrast, relatively few investigations have examined the physiological role of individual adipose depots by selectively removing them [9,10]. Depot-specific lipectomy offers a unique approach to assessing the contribution of adipose tissue-derived signals to cardiac growth, function, and stress adaptation, independent of obesity [11,12].

In the present study, we investigated the impact of removing visceral (VAT; gonadal fat) or subcutaneous (SAT; inguinal fat) white adipose tissue (WAT) on cardiac morphology and function, circulating adipokines, and myocardial gene expression in male and female mice. We also tested whether these baseline alterations influence the cardiac response to cardiometabolic stress by combining angiotensin II infusion with a high-fat diet, a model that exhibits several features of HFpEF [13]. Finally, we evaluated the contribution of ovarian hormones using ovariectomized female mice. We hypothesized that distinct adipose depots contribute to the maintenance of cardiac physiology and function and influence susceptibility to pathological stress in a sex-dependent manner.

## 2. Materials and Methods

### 2.1. Animals

**Figure 1:**
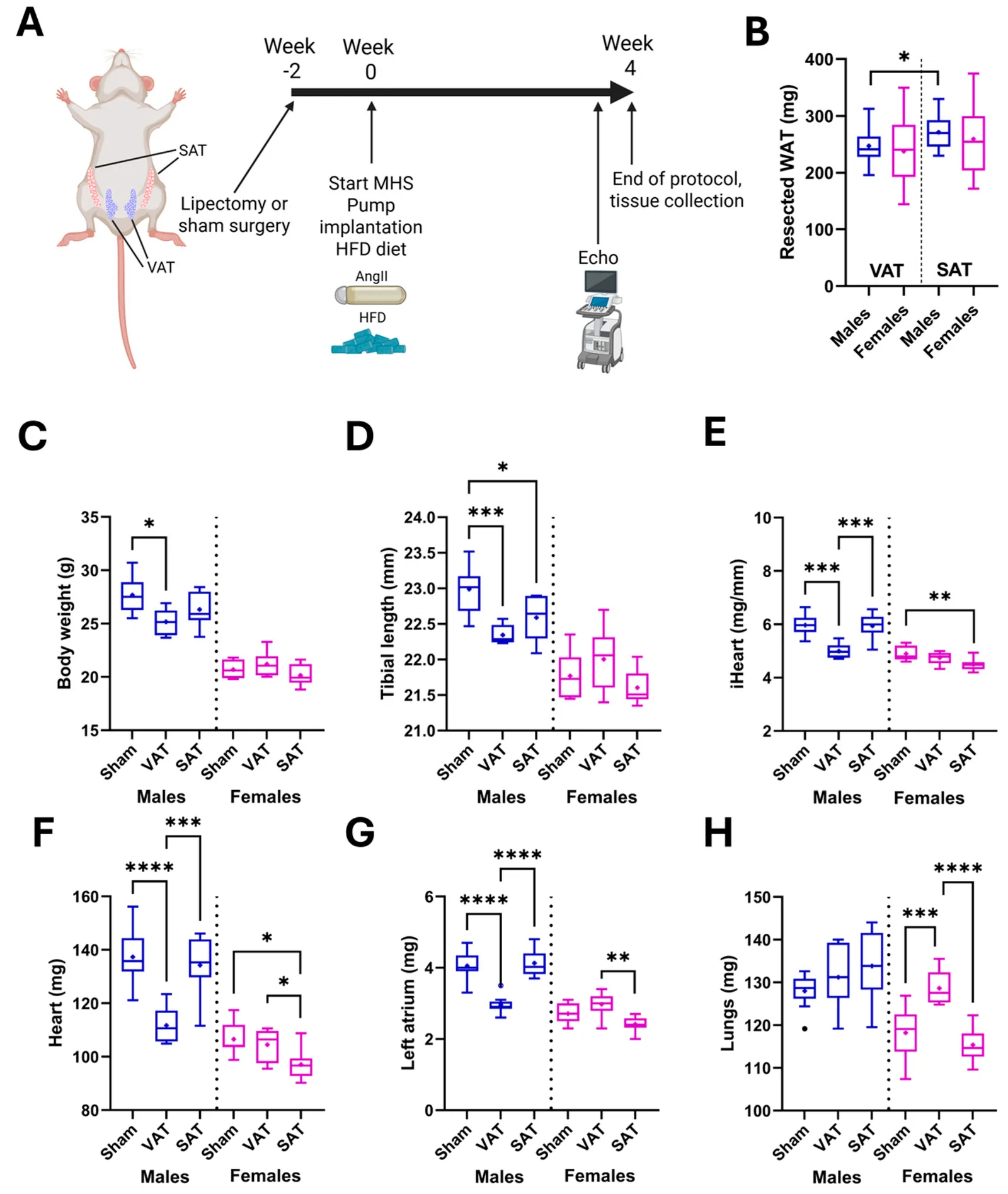
Effects of lipectomy on the mouse body and cardiac morphology. (**A**) Schematic representation of the experimental design (Created in BioRender. Couet, J. (2026) https://BioRender.com/n6l5lju). Seven-week-old C57BL/6J male (blue) and female (purple) mice were divided into 3 groups. Control mice underwent either a sham surgical procedure (Sham), bilateral ablation of the gonadal visceral fat depots (VAT) or ablation of the inguinal subcutaneous fat depots (SAT). Half of these mice received a standard diet for the following 10 weeks. The MHS (AngII + HFD) group received a standard diet for 4 weeks starting 2 weeks after surgery. (**B**) Resected visceral or subcutaneous adipose tissue weight, respectively, in male (blue, left) or female (fuchsia, right) animals. (**C**) Body weight. (**D**) Tibial length. (**E**) Indexed heart weight for tibial length (iHeart). (**F**) Heart weight. (**G**) Left atrial weight and (**H**) lungs wet weight. Results are expressed as the mean ± standard error of the mean (SEM)—one-way ANOVA followed by the Holm–Sidak post-test. *: *p* < 0.05, **: *p* < 0.01, ***: *p* < 0.001 and ****: *p* < 0.0001 between indicated groups (n = 7–8 mice/group).

C57BL/6J male and female 6-week-old mice were purchased from the Jackson Laboratory (Bar Harbor, ME, USA). Mice were housed (3–4 mice/cage) on a 12 h light–12 h dark cycle with free access to food and water. The Université Laval animal protection committee approved the protocol, and we followed the recommendations of the Canadian Council on Laboratory Animal Care (#2023-1250). We randomly assigned mice to the experimental groups. Figure 1A schematizes the general experimental design. Experienced technicians monitored the mice’s health and behaviour daily throughout the protocol, and weighed the animals weekly. As described before [12], metabolic and hypertensive stress (MHS) mice were implanted with an osmotic minipump to provide a continuous infusion of angiotensin II (AngII; 1.5 mg/kg/day) (Sigma, St. Louis, MO, USA) for 28 days and fed a high-fat diet (HFD: 60% calories; Research Diets Cat. #D12492).

### 2.2. Surgeries

Mice were isoflurane-anesthetized, had their abdominal fur shaved, and had betadine scrubbed over the incision site. In terms of VAT, a vertical midline incision along the lower abdomen was made to access gonadal adipose depots. In females, the adipose tissue surrounding the ovaries was dissected so as not to injure the gonads. Elsewhere, adipose depots were removed by blunt dissection. Mice that underwent the sham operation had their VAT mobilized but not removed. Absorbable sutures were used to close the body cavity while wound clips were used on the skin. Ovariectomy was performed following a similar procedure. For SAT surgeries, a midline subcutaneous (SC) incision was made; the skin was separated from the underlying musculature; both depots were removed; and the incision was closed with wound clips. Before surgery, all mice received an intraperitoneal injection of 20 mg/kg carprofen and subcutaneous injections of lidocaine and bupivacaine, with an additional dose (10 mg/kg) of carprofen the day after surgery.

### 2.3. Echocardiography

We performed echocardiography under isoflurane anesthesia as previously described [14].

### 2.4. RNA Isolation and Quantitative Real-Time Polymerase Chain Reaction

We extracted total RNA from LV tissue [13,15]. We used quantitative RT-PCR to quantify LV gene expression in at least six animals per group. Cyclophilin A (*Ppia*) served as the control housekeeping gene for cardiac gene analysis. The primers used are listed in Table S1.

### 2.5. Myocardial Fibrosis Evaluation

Myocardial samples were fixed, sliced in serial sections (10 μm thick), and stained with Picrosirius Red to assess the percentage of interstitial fibrosis as previously described [15]. We included perivascular fibrosis in the interstitial fibrosis estimate. We acquired images of an entire long-axis LV section for each animal using a wide-field microscope (Zeiss) and analyzed them in GIMP (GNU Image Manipulation Program; https://www.gimp.org/ accessed on 28 May 2026). Investigators performed both microscopy and image analysis while blinded to mouse identity. We determined the mean percentage of fibrosis. Briefly, we measured LV fibrosis in each section by calculating the ratio of stained (red) pixels to the total number of pixels representing cardiomyocytes and fibrosis. We measured the number of pixels corresponding to white sections (holes in the tissue). We subtracted this from the total number of pixels in the image to obtain the total number of pixels representing the myocardium. Measures were acquired using the ‘‘by colour select’’ tool in the software.

### 2.6. Cardiomyocyte Cross-Sectional Area

We visualized cardiomyocyte cross-sectional area (CSA) using immunofluorescent staining with wheat germ agglutinin-FITC (Sigma), as previously described [16].

For the ELISAs, mouse adiponectin (MRP300, #P520995), leptin (MOB00B, #P523603) and CCL2/MCP-1 (MJE00B, #P512456) ELISA kits were purchased from R&D Systems (BioTechne, Toronto, ON, Canada). We performed the assays according to the provider’s instructions.

### 2.7. Western Blot Analysis

We performed Western blotting as described previously [16]. Antibodies were diluted 1:1000 (PDK4 and p-PDH) or 1:250 (PDH1) in a TSB-T (Tris saline buffer þ Triton X-100) solution with 5% BSA. Phospho-PDH was obtained from Cell Signaling Technologies (Danvers, MA, USA; Cat. No. 31866), PDH (AB110416-1002), and PDK4 antibodies were from Abcam (AB214938) (Toronto, ON, Canada). The second antibody was diluted 1:2000 in TBS-T with 5% milk. We used Western Lightning Plus ECL (PerkinElmer, Woodbridge, ON, Canada) and ClarityMax ECL (Bio-Rad, Hercules, CA, USA) for membrane revelation with the Gel Doc system and Image Lab software (Bio-Rad, Version 6.1). We used the “Total-lane method” for normalization, adjusting for pre-blocking membrane exposure and a standard pool run on each gel.

### 2.8. Statistical Analysis

Data are presented as the mean ± standard error of the mean (SEM). We identified outliers using the ROUT method (Q = 1%) in GraphPad Prism (v11.0). We assumed normality based on sample size and distribution consistency. Comparisons between two groups were performed using unpaired Student’s *t*-tests. We used one-way ANOVA, followed by the Holm–Sidak post hoc test, when we analyzed more than two groups for a single independent variable. For multiple group comparisons, two-way ANOVA followed by Fisher’s least significant difference (LSD) post hoc test was used. A *p*-value < 0.05 was considered statistically significant. For variables analyzed using two-way ANOVA, we evaluated interaction terms and reported them in the corresponding figures and tables.

## 3. Results

### 3.1. Removal of a White Adipose Depot in Mice Is Associated with Changes in Cardiac Morphology

As illustrated in Figure 1A, C57BL/6J male and female mice were lipopectomized (Lpx) at 7 weeks of age. We compared the visceral gonadal white adipose depot (VAT) and the subcutaneous inguinal depot (SAT) to assess their effects on cardiac morphology and function. We studied sham-operated animals (Sham) in parallel. Two weeks later, we initiated MHS in half of the animals, and the remaining half (Ctrl) continued on a standard low-fat diet. The amount of white adipose tissue was comparable between male and female animals (Figure 1B).

VAT removal was associated with lower body weight and slower body growth (tibial length) in males than in Sham animals. The male SAT group showed a slight reduction in tibial length. In contrast, VAT and SAT Lpx had no effects in female mice (Figure 1C,D).

Male VAT mice also displayed smaller hearts, whereas SAT males had no change in heart size or indexed heart weight. In females, SAT Lpx resulted in a smaller heart and VAT ablation had no effects (Figure 1E,F). Taken together, these results suggest a WAT depot and sex-dependent response of the heart to Lpx. A similar observation was made for the left atrium, which was smaller in the male VAT group (Figure 1G). Interestingly, lung wet weight was significantly increased in VAT females but not in the SAT animals. (Figure 1C).

**Figure 2:**
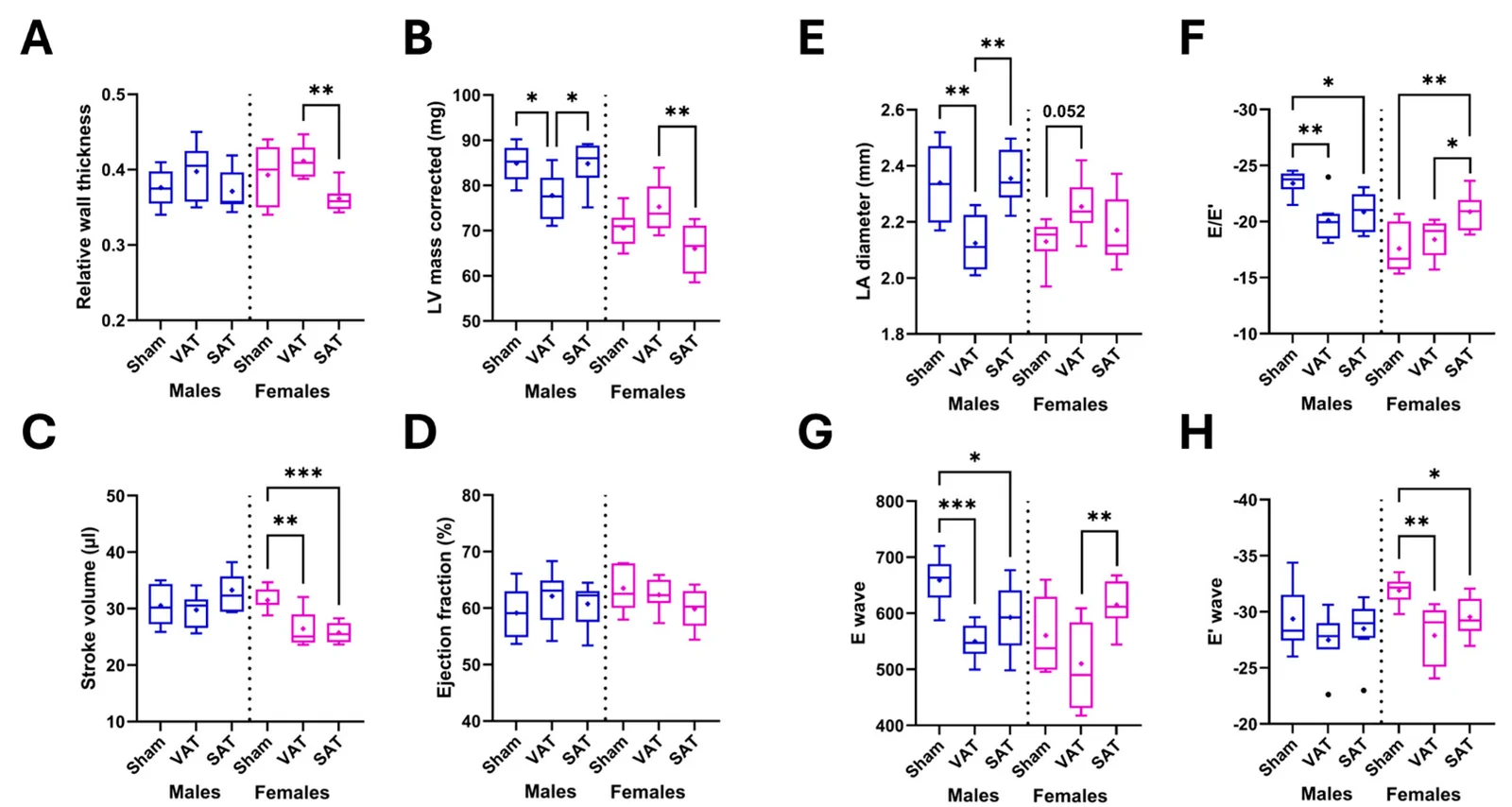
VAT or SAT lipectomy impacts echocardiography parameters in males and females. (**A**) Relative wall thickness (RWT; LV wall thickness/end-diastolic LV diameter). (**B**) Corrected LV mass as estimated by echo. (**C**) Stroke volume. (**D**) LV ejection fraction (EF; (end-diastolic volume (EDV)−end-systolic volume)/EDV * 100). (**E**) Left atrial (LA) diameter. (**F**) E/E’ ratio. (**G**) E wave. (**H**) E’ wave. Males: blue and left. Females: fuchsia and right. Results are expressed as mean ± standard error of the mean (SEM)—one-way ANOVA followed by the Holm–Sidak post-test. *: *p* < 0.05, **: *p* < 0.01, and ***: *p* < 0.00 between indicated groups (n = 7–8 mice/group).

Figure 2 shows cardiac morphological and functional changes induced by Lpx in control animals, as evaluated by echocardiography. As illustrated in Figure 2A, VAT or SAT Lpx was not associated with a general change in LV morphology as evaluated using the relative LV wall thickness (RWT; concentric or eccentric remodelling). However, in females, SAT and VAT differed significantly for this parameter, as did left ventricular mass (Figure 2B). This was emphasized by thicker LV walls in VAT females and thinner ones in the SAT group (Table S2). LV mass was lower in VAT males, as was heart weight. Stroke volume was unchanged in males but was lower in SAT females and, surprisingly, in the VAT group, too (Figure 2C). Lpx had no effects on the LV ejection fraction (Figure 2D).

We then measured several parameters related to diastolic function. In males, the LA diameter measured by echo was smaller in the VAT group. A strong tendency (*p* = 0.052) was observed for a larger LA diameter in the female SAT group (Figure 2E). Lpx in males was associated with lower E/E’ and E wave, whereas in females, SAT ablation increased these two parameters. The increased E/E’ in SAT females was also the product of a decreased E’ wave (Figure 2F–H). Additional echo data are available in Tables S2 and S3. Interestingly, SAT females had lower A and A’ waves, suggesting lower LA contractility. This was not observed in males.

**Figure 3:**
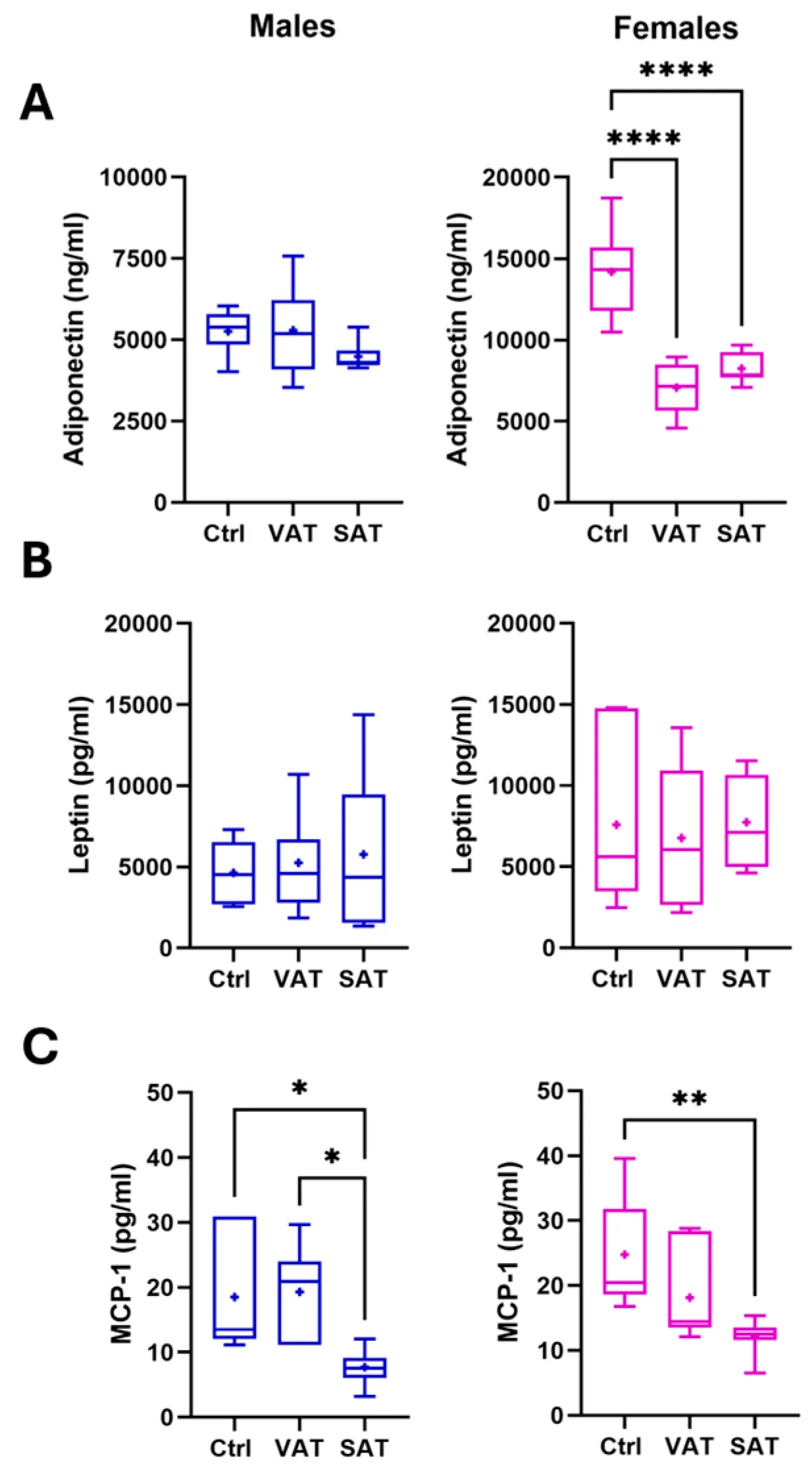
VAT or SAT lipectomy is associated with sex-dependent modulation of circulating adipokine levels. (**A**) Adiponectin. (**B**) Leptin. (**C**) MCP-1 (monocyte chemoattractant protein 1 or CCL2). Males: blue and left. Females: fuchsia and right. Results are expressed as mean ± standard error of the mean (SEM)—one-way ANOVA followed by the Holm–Sidak post-test. *: *p* < 0.05, **: *p* < 0.01, and ****: *p* < 0.0001 between indicated groups (n = 7–8 mice/group).

We measured the circulatory levels of two adipokines (adiponectin and leptin) and a cytokine (MCP-1 or CCL2) known to be secreted by the WAT in the plasma of the mice (Figure 3). As shown, Lpx significantly reduced adiponectin levels in females but not in males, suggesting different compensatory mechanisms between the sexes. Lpx did not alter leptin levels in our animals, but it reduced MCP-1 in SAT male and female groups.

### 3.2. WAT Removal Is Associated with Marked Changes in the Expression of Many Left Ventricle Genes

**Figure 4:**
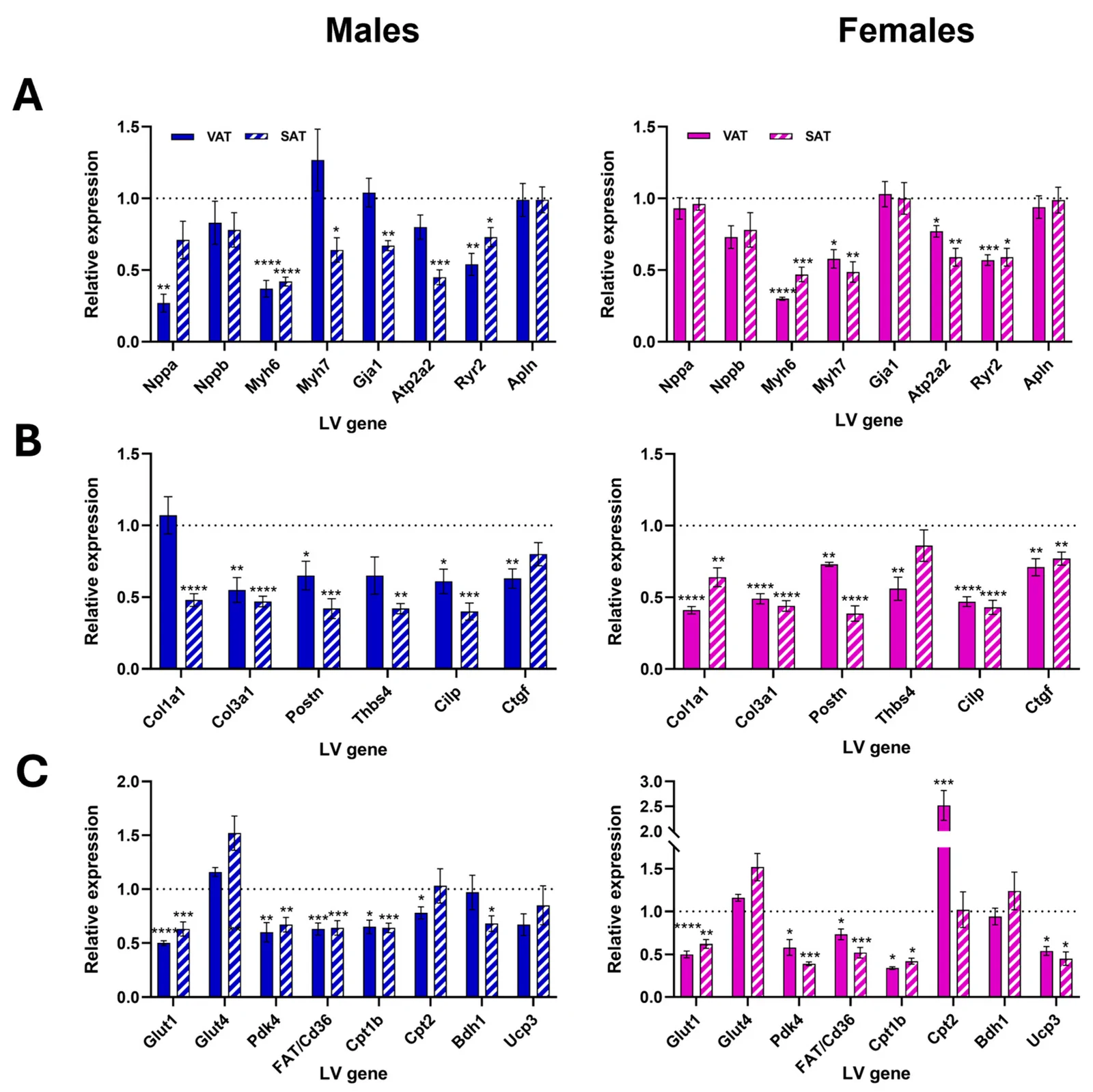
VAT or SAT lipectomy modulates a large group of LV genes in control male and female mice. (**A**) Modulation of LV genes associated with cardiomyocyte physiology. (**B**) LV genes associated with extracellular matrix remodelling. (**C**) Genes implicated in myocardial energy metabolism. The dotted line in each graph represents the relative expression of the indicated gene in sham-operated mice. Results are expressed as mean ± standard error of the mean (SEM)—Student’s *t*-test. *: *p* < 0.05, **: *p* < 0.01, ***: *p* < 0.001 and ****: *p* < 0.0001 between VAT or SAT animals and Sham mice (n = 6–8 mice/group).

Since WAT depot ablation was associated with sex- and depot-specific changes in cardiac growth, we investigated whether the Lpx also modulated LV gene expression. We studied three gene groups: basic markers of hypertrophy, physiology, and calcium handling (Figure 4A); genes related to the extracellular matrix (ECM) (Figure 4B); and genes involved in myocardial energy metabolism (Figure 4C).

As illustrated in Figure 4A, basal LV mRNA levels of many genes are lowered in VAT and SAT animals. *Myh6* and *Myh7* (in females) levels are both strongly reduced in the myocardium of Lpx mice in male and female mice. *Atp2a2* (Serca2) and *Ryr2* expression levels are also reduced in mice after Lpx. Genes related to ECM physiology and expressed by cardiac fibroblasts were almost all reduced in Lpx mice compared to sham animals, and this was observed for both sexes (Figure 4B). This pattern also applied to genes implicated in energy production. Glucose transporter 4 (Glut4) was a notable exception, as were Cpt2 and *Bdh1* (Figure 4C).

### 3.3. WAT Removal Effects on the Cardiac Morphological and Functional Response to a HFpEF-Inducing Stress Are Sex-Specific

**Figure 5:**
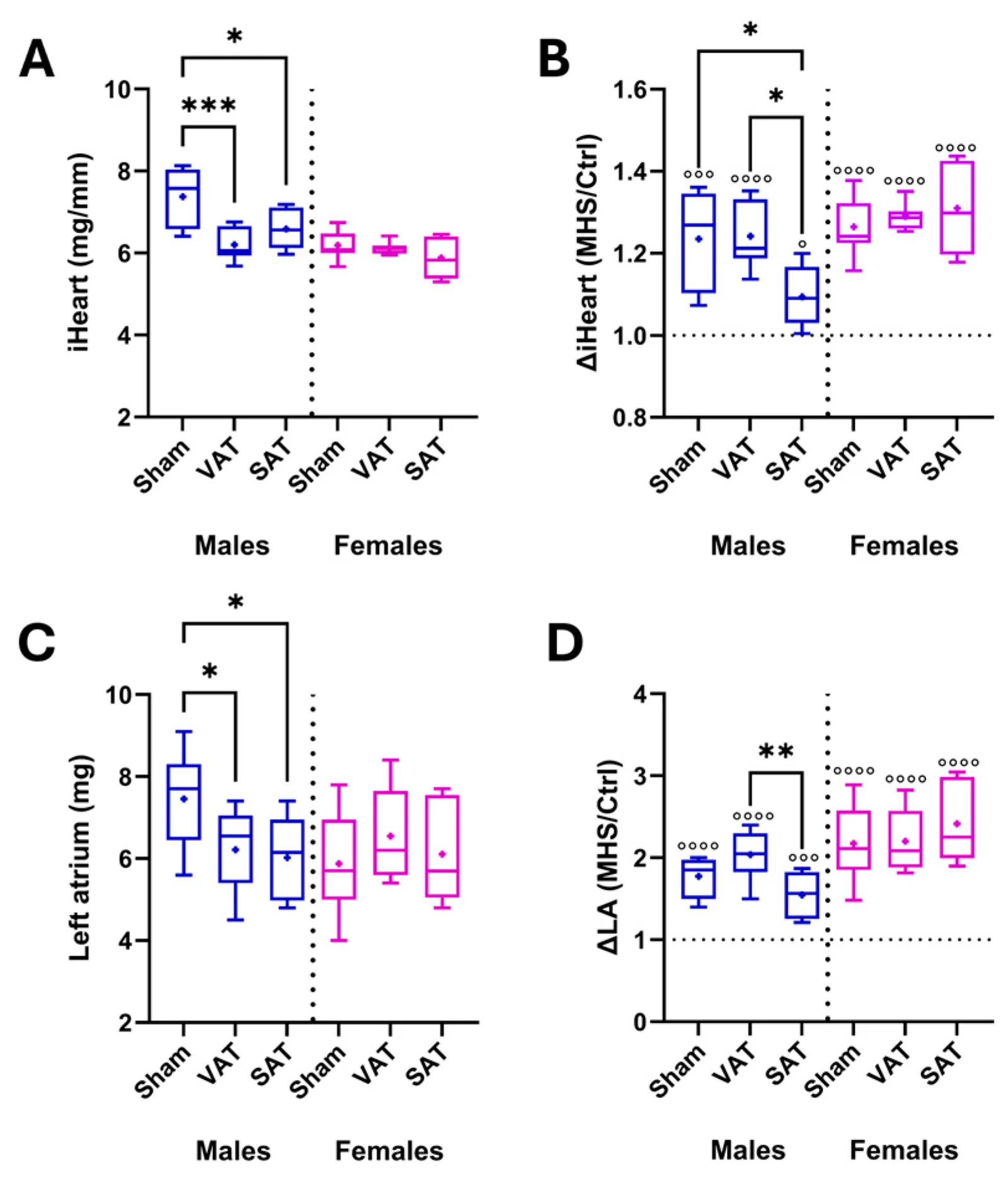
VAT or SAT lipectomy influence the hypertrophic response of the heart to the MHS in a fat depot- and sex-dependent manner. (**A**) Indexed heart weight of MHS mice. (**B**) Ratio of MHS over Ctrl iHeart. (**C**) LA weight of MHS mice. (**D**) Ratio of MHS over Ctrl LA weight. The dotted line in graphs (**B**,**D**) represents control animals. Males: blue and left. Females: fuchsia and right. Results are expressed as mean ± standard error of the mean (SEM)— one-way ANOVA followed by the Holm–Sidak post-test. *: *p* < 0.05, **: *p* < 0.01, and ***: *p* < 0.001 between indicated groups (n = 7–8 mice/group). Student’s *t*-test. °: *p* < 0.05, °°°: *p* < 0.001 and °°°°: *p* < 0.0001 between VAT or SAT animals and control (non-MHS) mice.

We recently developed a murine two-hit model of HFpEF that produces a cardiac phenotype similar in both sexes. Fed a high-fat diet, the mice received a continuous infusion of AngII via a subcutaneous osmotic minipump (MHS) for 4 weeks. This regimen produced a hypertrophic response averaging 25% in both sexes (Figure 5A,B). Interestingly, indexed heart weights were lower in male mice in the Lpx groups than in sham animals. This was not observed in females (Figure 4A). When reported to the heart weight of control animals, the heart weight gain caused by the MHS was similar in all groups except the male SAT animals, which showed a reduced hypertrophic response of only 9% (Figure 4B). Similarly, left atrial enlargement consequent to the MHS (Figure 4C) differed between male VAT and SAT groups, with the latter being lower. In females, LA enlargement was similar in all groups.

Figure S1 shows cardiac morphological and functional parameters evaluated by echocardiography, induced by the Lpx in MHS animals. As illustrated in Figure S1A, VAT Lpx was associated with increased concentric LV remodelling after MHS. This was the only statistically significant difference recorded between sham-operated and Lpx mice of both sexes.

A similar pattern was observed for LV gene expression in MHS mice (Figure S2), as the modulation pattern in HFpEF-like mice was relatively similar in sham and Lpx mice. We then tested whether changes in LV gene expression after Lpx were associated with protein-level modulation. Pyruvate dehydrogenase kinase 4 phosphorylates the pyruvate dehydrogenase (PDH) complex, inactivating it. Since Lpx was associated with lower *Pdk4* mRNA levels, we measured whether the phosphorylated PHD (pPDH) content was also decreased. As observed in Figure S3, pPDH content was reduced in the LV of female mice but not males. We previously showed that MHS was associated with higher levels of PDH phosphorylation [15]. We also observed this in our mice, and Lpx had no impact.

Interstitial and perivascular myocardial fibrosis were also increased in MHS mice, as previously described. Interestingly, WAT removal increased baseline levels of interstitial fibrosis but not perivascular fibrosis. As expected, MHS increased myocardial fibrosis, and WAT removal was associated with even higher collagen levels in females (Figure S4).

We also measured cardiomyocyte area (CSA) in our mice. We noticed that Lpx was associated with larger CSA in control female mice. By contrast, MHS was associated with a smaller hypertrophic response in Lpx females. This was not observed in males (Figure S5).

**Figure 6:**
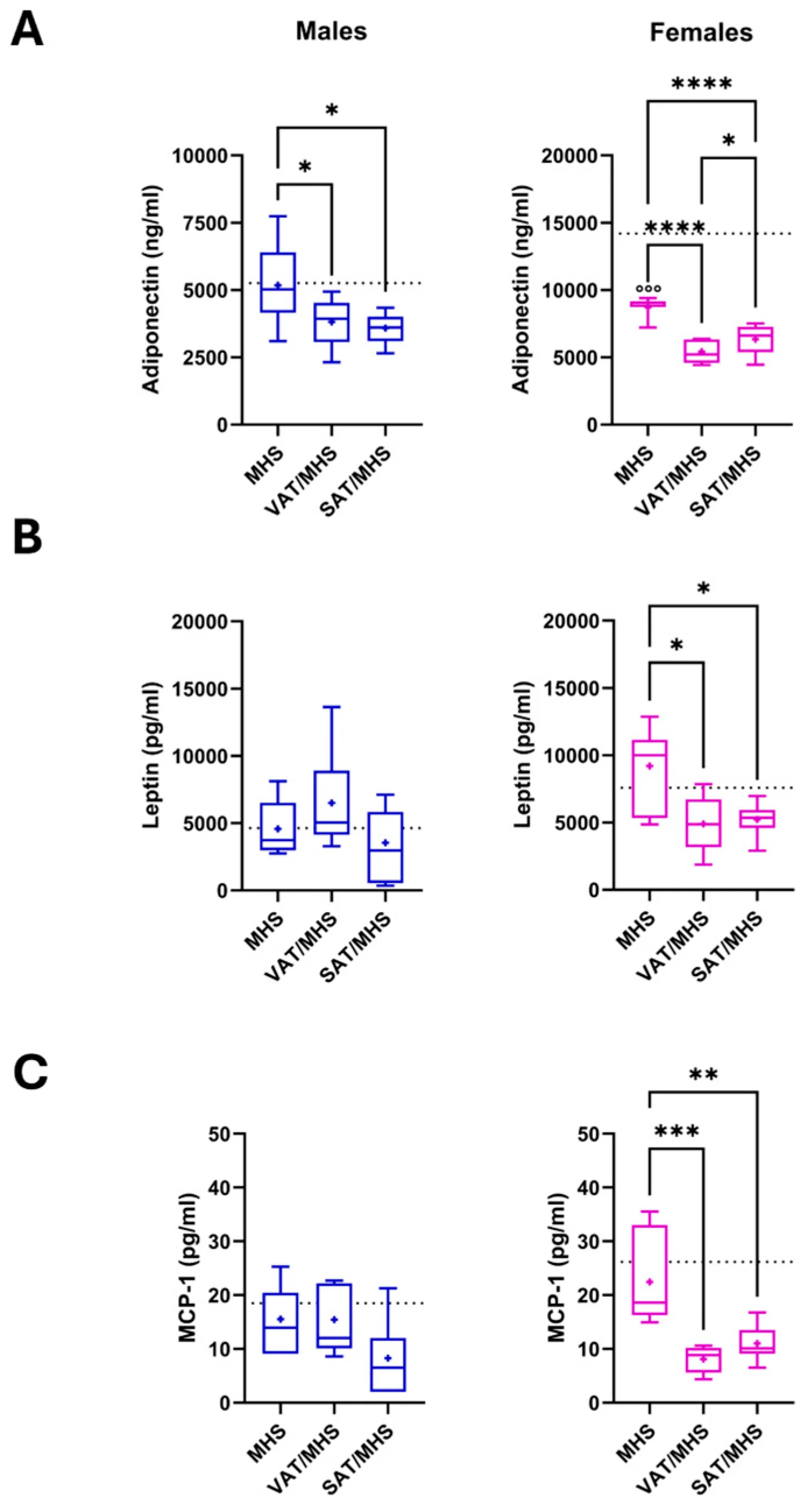
VAT or SAT lipectomy is associated with sex-dependent modulation of adipokine levels in the circulation of MHS mice. (**A**) Adiponectin. (**B**) Leptin. (**C**) MCP-1 (monocyte chemoattractant protein 1 or CCL2). Males: blue and left. Females: fuchsia and right. Results are expressed as mean ± standard error of the mean (SEM)—one-way ANOVA followed by the Holm–Sidak post-test. *: *p* < 0.05, **: *p* < 0.01, **: *p* < 0.001 and ****: *p* < 0.0001 between indicated groups. °°°: *p* < 0.001 between MHS and controls. (n = 7–8 mice/group).

We then measured circulatory levels of adiponectin, lectin, and MCP-1 (Figure 6). As illustrated in Figure 6A, adiponectin levels were significantly reduced by Lpx in male and female mice. Interestingly, MHS was associated with lower adiponectin levels in sham females but not in males. Leptin and MCP-1 levels were unchanged in males, but Lpx in MHS females significantly reduced these levels (Figure 6B,C).

### 3.4. VAT Removal Effects in Ovariectomized Females Are Associated with Larger Hearts and Lungs

Because we observed several sex-related differences in the cardiac response to WAT removal, we investigated whether ovarian hormones modulate cardiac parameters after VAT Lpx. VAT removal in intact females was not associated with marked changes in mouse body or cardiac morphology. In ovariectomized (Ovx) females, VAT removal resulted in increased body growth (tibial length) and larger hearts (Figure S6A–D). The left atrium weight was not modified by VAT removal (Figure S6E,F). In MHS ovx females, VAT was associated with increased body weight and larger hearts, but the LA weight was similar to that of the sham animals (Figure S6E,F). MHS reduced adiponectin levels, but VAT removal did not (Figure S7).

**Figure 7:**
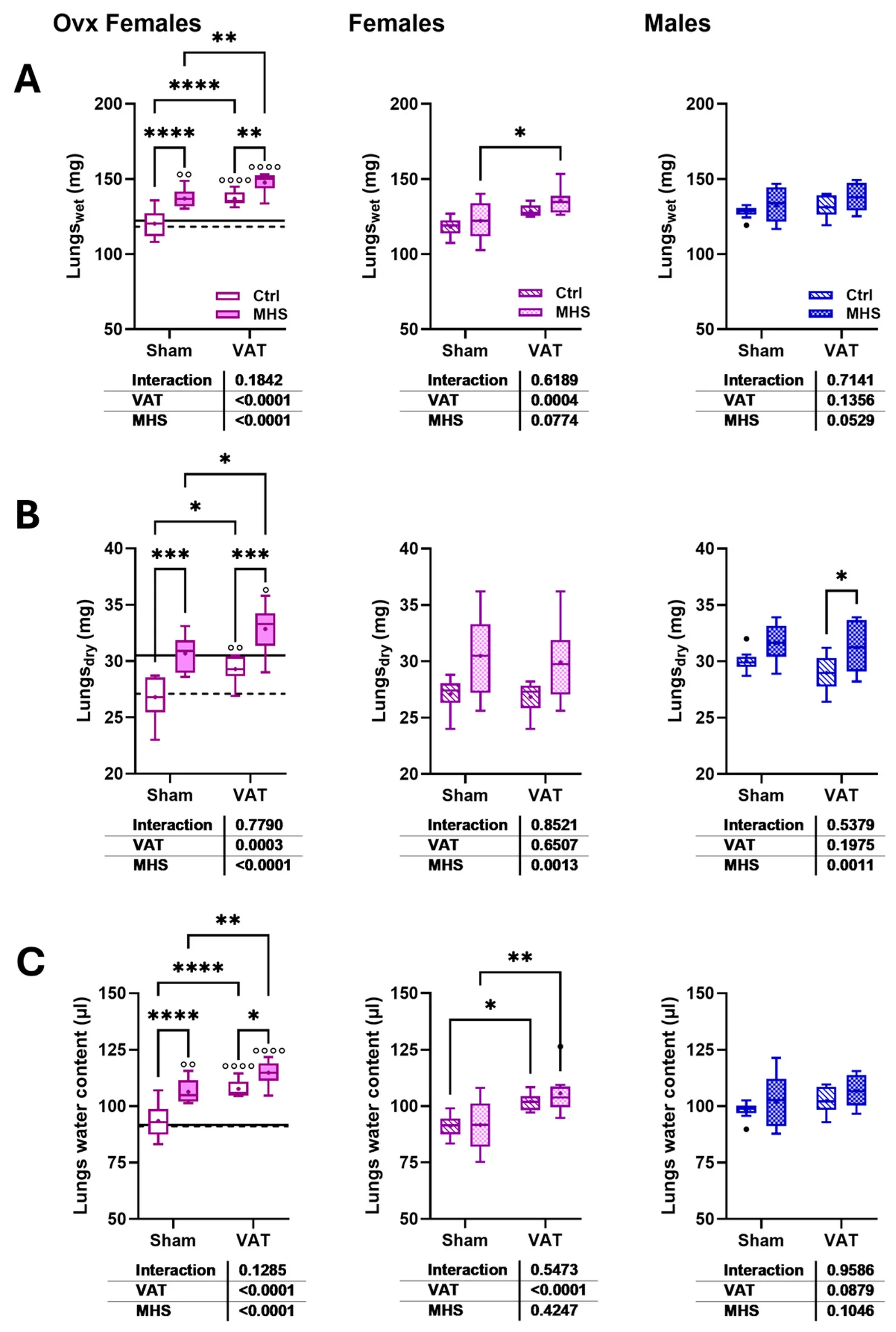
Ovarian hormone depletion exacerbates pulmonary alterations associated with visceral adipose tissue removal. Lung wet weight (**A**), lung dry weight (**B**), and estimated lung water content (**C**) in ovariectomized (Ovx) females, intact females, and males subjected to sham surgery or visceral adipose tissue (VAT) lipectomy and subsequently exposed to control conditions (Ctrl) or metabolic and hypertensive stress (MHS). Horizontal dashed lines indicate the mean value of the corresponding control group. Box plots represent median, interquartile range, and minimum-to-maximum values; individual data points are shown. Statistical analyses were performed using two-way ANOVA with VAT lipectomy and MHS as factors. Corresponding *p* values for interaction and main effects are shown below each graph. * *p* < 0.05, ** *p* < 0.01, *** *p* < 0.001, **** *p* < 0.0001. °: *p* < 0.05, °°: *p* < 0.01 and °°°°: *p* < 0.0001 between Ovx groups and corresponding non-Ovx groups. Abbreviations: Ovx, ovariectomized; VAT, visceral adipose tissue; Ctrl, control; MHS, metabolic and hypertensive stress.

We observed that VAT removal also increased lung wet weight in intact females but not males (Figure 1H). This VAT removal effect was clearer in Ovx females (Figure 7A). To determine whether this difference resulted from additional pulmonary tissue or water content, we dried lungs overnight at 80 °C and weighed them again. This increase in wet weight in the VAT group was due to both increased pulmonary tissue and water content (Figure 7B,C). In intact females, lung dry weight was unaffected in the VAT group, whereas water content was increased. This was not observed in males.

We previously showed that 4 weeks of MHS was not associated with lung congestion (increased wet lung weight) and that this stress had to be extended to 8 weeks to increase this parameter. Here, we observed that 4 weeks of MHS was associated with increased lung tissue content in females (Ovx or not) and males; however, water content increased only in Ovx females after MHS.

We also evaluated expression for the same panel of LV genes in Ovx females ± VAT removal (Tables S4–S6). The VAT group displayed lower mRNA levels of *Myh6*, *Serca2*, *Ryr2*, and *Apln*. Genes associated with ECM remodelling were identical to sham controls, except for Col1a1, and those related to myocardial metabolism remained unchanged. After MHS, we observed reduced modulation of *Nppa*, *Nppb*, *Serca2*, *Ryr2*, and *Apln* in VAT mice compared with sham mice. This was not the case for ECM genes, except for *Ctgf*. *Cpt2* and *Ucp3* were less upregulated in VAT females.

## 4. Discussion

### 4.1. White Adipose Tissue Helps Maintain the Cardiac Phenotype

This study shows that WAT helps maintain normal cardiac structure and function in mice. Selective removal of either visceral or subcutaneous adipose depots caused significant alterations in cardiac morphology, diastolic function, circulating adipokine levels, and myocardial gene expression despite the absence of overt cardiovascular disease. These observations indicate that adipose depots are not merely passive energy-storage compartments but actively regulate cardiac physiology. Interestingly, VAT removal was associated with a modest reduction in tibial length in males. Because lipectomies were performed during a period of ongoing somatic growth, these findings suggest that adipose tissue-derived signals may contribute not only to cardiac growth but also to whole-body development. Most of the literature in the field has concentrated on body weight, not growth [17]. Whether similar effects occur in fully mature animals remains unknown.

One of the most striking findings is that depot-specific lipectomy modified cardiac growth in opposite directions depending on the adipose depot and sex studied. Removal of VAT reduced heart size and left atrial mass in males, whereas SAT removal reduced cardiac mass in females. These structural changes were accompanied by alterations in several echocardiographic parameters, including left ventricular mass, atrial dimensions and indices related to diastolic physiology. The observation that these effects occurred in otherwise healthy animals strongly suggests that AT-derived signals normally contribute to the physiological regulation of cardiac growth and function.

Further supporting this concept, Lpx induced extensive changes in the expression of genes involved in calcium handling, contractile function, extracellular matrix turnover and energy metabolism. The coordinated reduction in Myh6, Atp2a2, and Ryr2 expression, along with multiple extracellular matrix genes, points to broad reprogramming of myocardial physiology following AT removal. These molecular changes were disproportionate to the relatively modest structural alterations observed, suggesting that AT exerts a continuous regulatory influence on the myocardium even without pathological stress. Lpx also influenced basal interstitial fibrosis in control mice. This was unexpected and suggests that WAT controls the myocardial ECM.

### 4.2. Adipose–Cardiac Interactions Depend Heavily on Adipose Depot, Sex, and Ovarian Hormones

A second major finding is that AT’s influence on the heart is highly context-dependent. VAT and SAT did not exert identical effects, and removing them often produced distinct or even opposite cardiac responses. These observations align with well-established biological differences between adipose depots in developmental origin, endocrine profile, and metabolic activity.

The markedly higher circulating adiponectin concentrations in female mice than in males are consistent with previous reports in both rodents and humans [18,19]. These sex differences have been attributed to sex hormones influencing adipose tissue biology and adipokine secretion and may contribute to sex-specific cardiovascular phenotypes.

Several findings suggest that sex is another important determinant of WAT-cardiac communication. For example, visceral lipectomy reduced left ventricular mass and left atrial size in males, whereas females exhibited more pronounced changes after subcutaneous fat removal. Similarly, indices of diastolic function responded differently to lipectomy in both sexes. E/E’ decreased in males after AT removal but increased in females after SAT ablation, suggesting fundamentally different roles of adipose-derived signals in the regulation of ventricular filling pressures and relaxation. These observations are consistent with the notion that AT distribution and endocrine activity contribute to well-described sex differences in cardiovascular physiology and disease susceptibility.

We investigated whether the reduction in LV Pdk4 gene expression in Lpx animals was correlated with a decrease in PDH phosphorylation in this tissue. The reduction in myocardial *Pdk4* transcript levels was accompanied by lower PDH phosphorylation in females, suggesting that some transcriptional changes induced by lipectomy translate into measurable metabolic alterations. This was not the case for males. This is an important reminder that the marked effects of Lpx on LV gene expression do not directly reflect the situation at the protein level, and other factors are at play.

The ovariectomy experiments support a role for ovarian hormones in modulating these interactions. Although VAT removal produced relatively limited morphological changes in intact females, its effects became more apparent after ovarian hormone deprivation. In particular, VAT-lipectomized ovariectomized females had larger hearts and a distinct pulmonary phenotype, supporting a three-way interaction among adipose depots, the myocardium, and ovarian hormones. Because we did not measure circulating estrogen levels, we cannot exclude indirect effects of VAT removal on ovarian function in intact females. Although care was taken to preserve ovarian integrity during surgery, the intimate physiological relationship between visceral adipose tissue and reproductive organs deserves further investigation.

The pulmonary findings in ovariectomized females warrant particular attention. VAT removal was associated with increases in lung wet weight, dry weight, and estimated water content, with these effects becoming more pronounced after ovariectomy. These changes may reflect altered pulmonary fluid handling or increased susceptibility to pulmonary congestion, suggesting that adipose tissue’s physiological influence extends beyond the myocardium. Given the importance of pulmonary congestion in clinical heart failure, these observations warrant further investigation. The pulmonary phenotype observed in ovariectomized VAT-Lpx females is intriguing, suggesting that adipose tissue’s physiological influence extends beyond cardiac morphology and may contribute to the regulation of pulmonary fluid homeostasis. These findings are particularly intriguing given the high prevalence of HFpEF among post-menopausal women and the recognized contribution of ovarian hormone loss to disease susceptibility [1,20,21]. Although we did not measure pulmonary pressures in the present study, the increased lung water content observed in ovariectomized VAT-Lpx mice raises the possibility that visceral adipose tissue and ovarian hormones interact to preserve cardiopulmonary homeostasis. Although the mechanisms remain unclear, the findings are consistent with a protective interaction between ovarian hormones and VAT. Given the central role of pulmonary congestion in HFpEF pathophysiology, this observation warrants further investigation.

### 4.3. Baseline Alterations Induced by Lipectomy Influence Adaptation to Cardiometabolic Stress

Because lipectomy altered the baseline cardiac phenotype, we hypothesized that these differences could influence adaptation to pathological stress. Combined angiotensin II infusion and high-fat feeding induced the expected hypertrophic response in both sexes [13,16]. However, the magnitude and characteristics of this response varied with adipose depot status. In males, SAT lipectomy attenuated cardiac hypertrophy and reduced left atrial enlargement, whereas females exhibited a different response pattern. These findings indicate that pre-existing alterations in the cardiac phenotype can modify the subsequent adaptation to cardiometabolic stress.

Interestingly, the effects of lipectomy on the HFpEF-like phenotype were generally more modest than those observed under baseline conditions. One possible explanation is that the MHS itself substantially modifies the physiology of the remaining adipose depots. Angiotensin II promotes lipolysis and induces profound changes in adipose tissue biology [22], whereas high-fat feeding alters adipose tissue composition and endocrine activity. We previously observed that the MHS markedly affected the circulatory microRNA (miR) profile [23]. Because adipose tissue contributes significantly to the circulating pool of extracellular vesicles and microRNAs (miRs), the MHS may modify signals that affect AT-heart communication. Consequently, some baseline differences induced by depot removal may be attenuated once animals are exposed to cardiometabolic stress. This suggests that adipose depots may not function primarily as determinants of disease severity but rather as modulators of myocardial susceptibility and adaptation. In this context, the MHS model can be viewed as revealing the functional importance of adipose-cardiac interactions that are already present under physiological conditions.

Recent work has shown that visceral adipose tissue can actively contribute to HFpEF progression by secreting extracellular vesicles, as VAT resection attenuated disease severity. In contrast, VAT transplantation aggravated it in a murine HFpEF model [24]. Our findings complement these observations. Rather than focusing on a single mechanistic pathway, we show that adipose depots exert broad physiological effects on cardiac structure, function and gene expression even in the absence of overt disease. Together, these studies support a functional adipose–cardiac axis and suggest that adipose tissue may influence both normal myocardial function and adaptation to pathological stress.

### 4.4. Potential Mechanisms Mediating Adipose-Cardiac Communication

The present study was not designed to identify a single mediator responsible for the observed effects. Nevertheless, several observations support a complex endocrine communication axis linking adipose tissue and the heart. Removal of specific adipose depots altered circulating adiponectin and MCP-1 levels and produced broad transcriptional changes in the myocardium. These findings support the hypothesis that adipose-derived factors contribute to the regulation of cardiac physiology.

Interestingly, circulating leptin levels remained largely unchanged following depot-specific lipectomy despite the substantial reduction in adipose tissue mass. Although we did not investigate the remaining adipose depots, compensatory adaptations in residual fat stores may have helped maintain circulating leptin concentrations [25].

A single adipokine is unlikely to explain the entire observed phenotype. White adipose tissue secretes a wide variety of signalling molecules, including adipokines, cytokines, lipids, extracellular vesicles and microRNAs, many of which may exert overlapping or complementary biological actions [19,26]. Recent studies have shown that adipose-derived extracellular vesicles can influence cardiac hypertrophy and fibrosis [9,12,24]. At the same time, our previous work demonstrated profound alterations in circulating microRNA profiles in mice exposed to cardiometabolic stress [23]. Together, these observations support a model in which adipose depots regulate cardiac physiology through multiple converging systemic pathways rather than through a single dominant mediator. Further studies are needed to elucidate the mechanisms by which WAT exerts control over cardiac physiology. WAT transplantation studies could also help understand this interplay by restoring normal cardiac physiology after Lpx [27,28,29,30].

## 5. Study Limitations

A major limitation of this study is that selective removal of adipose depots does not identify the specific mediators responsible for the observed cardiac effects. White adipose tissue secretes a broad range of bioactive molecules, including adipokines, cytokines, lipid mediators, extracellular vesicles and microRNAs. These experiments were not designed to distinguish among these potential mechanisms. Future studies are needed to characterize the molecular signals responsible for adipose-cardiac communication.

We did not assess compensatory hypertrophy or endocrine remodelling of the remaining adipose depots following lipectomy. These adaptations may have contributed to maintained circulating leptin levels and may have influenced cardiac responses observed later in the study.

Our study was performed in young mice whose cardiac growth was not fully complete. In contrast, HFpEF predominantly affects older individuals and is strongly influenced by age-dependent physiological changes. Whether similar adipose-cardiac interactions occur during ageing remains to be determined.

We studied gonadal visceral adipose tissue and inguinal subcutaneous adipose tissue, which may not fully represent the physiological diversity of all adipose depots.

The HFpEF-like phenotype induced by the combination of angiotensin II and high-fat feeding remained relatively moderate [13]. Although significant cardiac hypertrophy and atrial remodelling were observed, overt heart failure manifestations were limited. Consequently, the impact of adipose depot removal on more advanced disease stages remains uncertain.

Although we observed extensive changes in myocardial gene expression, protein validation was limited to PDH phosphorylation. Consequently, the functional significance of many transcriptional changes remains inferential and should be confirmed in future studies.

Ovariectomy provides a useful experimental model of ovarian hormone deprivation but does not fully reproduce the complex physiological changes associated with natural menopause. Therefore, extrapolate these findings to postmenopausal women with caution.

Finally, our objective was not to identify a single causal pathway but to determine whether individual adipose depots contribute to cardiac homeostasis and stress adaptation. This integrative physiological approach necessarily limits mechanistic conclusions but provides a broader understanding of adipose-cardiac interactions.

## 6. Conclusions

Our results do not support a simplistic view that adipose tissue is either beneficial or detrimental to the cardiovascular system. Instead, they suggest that distinct adipose depots help maintain cardiac homeostasis under physiological conditions, while potentially contributing to pathological remodelling under specific cardiometabolic stresses. This interpretation aligns with emerging evidence implicating adipose tissue-derived extracellular vesicles and other endocrine mediators in regulating cardiac remodelling and HFpEF progression.

These findings identify white adipose tissue as an active regulator of cardiac homeostasis and reveal that the cardiac consequences of adipose tissue loss are strongly influenced by depot specificity, biological sex and ovarian hormone status.

## Data Availability

The original contributions presented in this study are included in the article/Supplementary Materials. Further inquiries can be directed to the corresponding author.

## Supplementary Materials

The following supporting information can be downloaded at https://www.mdpi.com/article/10.3390/biom16091364/s1. Figure S1: VAT or SAT lipectomy has little effect on echocardiography parameters in MHS males and females compared to controls. A. RWT left and right, B. DRWT, C. Corrected LV mass, D. DLV mass, E. Stroke volume (SV), F. DSV, G. LV ejection fraction, H. DE/E’ ratio. Males: blue and left. Females: fuchsia and right. Results are expressed as mean ± standard error of the mean (SEM); one-way ANOVA followed by the Holm-Sidak post-test. *: *p* < 0.05, **: *p* < 0.01, and ***: *p* < 0.00 between indicated groups (n = 7–8 mice/group); Figure S2: VAT or SAT lipectomy result in the modulation of LV genes in MHS male and female mice. A. Modulation of LV genes associated with cardiomyocyte physiology. B. LV genes associated with extracellular matrix remodelling; C. Genes implicated in myocardial energy metabolism. The dotted line in each graph represents the relative expression of the indicated gene in control mice. Results are expressed as mean ± standard error of the mean (SEM); one-way ANOVA followed by the Holm-Sidak post-test. *: *p* < 0.05, **: *p* < 0.01, and ***: *p* < 0.001 between VAT or SAT groups and Sham (n = 7–8 mice/group). Student’s *t*-test. °: *p* < 0.05, °°°: *p* < 0.001 and °°°°: *p* < 0.0001 between VAT or SAT animals and control (non-MHS) mice; Figure S3: Impact of lipectomy on phosphorylation levels of the LV pyruvate dehydrogenase (PDH). A. Representative images of immunoblots of sham and Lpx male and female mice of phospho-PDH (pPDH) and PDH contents. B. Protein contents in control animals. C. Protein contents in MHS mice. Data are presented as mean ± SEM (n = 6–8 per group). One-way ANOVA followed by the Holm-Sidak post-test. *: *p* < 0.05, **: *p* < 0.01, and ***: *p* < 0.001 between indicated groups. °: *p* < 0.05, °°: *p* < 0.01, °°°: *p* < 0.001 and °°°°: *p* < 0.0001 between indicated MHS and corresponding control group (n = 7–8 mice/group); Figure S4: Lipiectomy is not associated with different levels of myocardial interstitial fibrosis in MHS mice. A. Representative images of picrosirius red staining of control male and female heart sections. B. Representative images of picrosirius red staining of MHS male and female heart sections. Interstitial fibrosis levels in control (left) and MHS (right) animals. Up. Males and females (bottom). Females (D) Myocardial fibrosis (picrosirius red staining). Data are presented as mean ± SEM (n = 7–8 per group). One-way ANOVA followed by the Holm-Sidak post-test. *: *p* < 0.05, **: *p* < 0.01, and ***: *p* < 0.001 between indicated groups (left graphs) and MHS vs. Control groups (right) (n = 5–7 mice/group); Figure S5: VAT or SAT lipectomy is associated with increased cardiomyocyte cross-sectional area in females, but not males. A. Cardiomyocyte cross-sectional area (CSA), B. DCSA. C. Representative images of WGA-FITC staining from LV sections of various indicated groups (Males). D. Females. Bar: 50µm. Data are presented as mean ± SEM (n = 7–8 per group). One-way ANOVA followed by the Holm-Sidak post-test. *: *p* < 0.05, **: *p* < 0.01, ***: *p* < 0.001 and, ****: *p* < 0.0001 between indicated groups (n = 6–8 mice/group); Figure S6: Removal of ovarian hormones is not associated with major differences in the mouse body and cardiac morphology in females with VAT lipectomy. A. Body weight. B. Tibial length, C. Heart weight, D. Indexed heart weight (iHeart). E. Left atrial weight and F. Indexed LA weight (iLA). The dotted line on the graphs represents the mean of the indicated parameter in control non-Ovx females, and the solid line in MHS mice. Results are expressed as mean ± standard error of the mean (SEM); two-way ANOVA followed by Fisher’s Least Significant Difference (LSD) test. The two-way ANOVA results are presented below the graph for each variable and the Interaction (MHS × VAT). *: *p* < 0.05, **: *p* < 0.01, ***: *p* < 0.001 and ****: *p* < 0.0001 between indicated groups (n = 7–8 mice/group). °°: *p* < 0.01, between indicated MHS and corresponding control group (n = 7–8 mice/group); Figure S7: VAT lipectomy modulates adiponectin levels in the circulation of ovariectomized (Ovx) mice. The dotted line represents levels in non-Ovx control females. Results are expressed as mean ± standard error of the mean (SEM); two-way ANOVA followed by Fisher’s Least Significant Difference (LSD) test. The two-way ANOVA results are presented below the graph for each variable and the Interaction (MHS × VAT). **: *p* < 0.01, and ***: *p* < 0.001 between indicated groups (n = 7–8 mice/group). Student’s *t*-test. °: *p* < 0.05, between Ovx animals and non-OVX control mice; Table S1: Sequences of primers used in this study; Table S2: Echo data in male and female mice after visceral (VAT) or subcutaneous white adipose tissue (SAT) depot lipectomy. Echo exams as described in the Methods section. IVSd: diastolic interventricular septum; PWd: diastolic posterior wall thickness; ESD: end-systolic LV diameter; LV mass; HR: heart rate; CO: cardiac output; EDV: end-diastolic volume. Results are expressed as the mean ± standard error of the mean (SEM). *p* values were calculated using the Holm–Sidak post hoc test after a one-way ANOVA. a: *p* < 0.05 vs sham group; b: *p* < 0.01; c: *p* < 0.001; d: *p* < 0.0001. e: *p* < 0.05 between lipectomy groups; f: *p* < 0.01; g: *p* < 0.001; and h: *p* < 0.0001; Table S3. Diastolic echo data in male and female mice after visceral (VAT) or subcutaneous white adipose tissue (SAT) depot lipectomy. Controls (C). Vehicle (Veh). Echo exams as described in the Methods section. A-wave velocity, E’ wave velocity, and A’ wave velocity. Results are expressed as the mean ± standard error of the mean (SEM). *p* values were calculated using the Holm–Sidak post hoc test after a one-way ANOVA. a: *p* < 0.05 vs sham group; b: *p* < 0.01; c: *p* < 0.00; d: *p* < 0.0001. e: *p* < 0.05 between lipectomy groups; f: *p* < 0.01; g: *p* < 0.001; h: *p* < 0.0001; Tables S4–S6: VAT or SAT lipectomy modulation of LV genes in OVX mice. A. Modulation of LV genes associated with cardiomyocyte physiology. B. LV genes associated with extracellular matrix remodelling; C. Genes implicated in myocardial energy metabolism. The dotted line in each graph represents the relative expression of the indicated gene in control mice. Results are expressed as the mean ± standard error of the mean (SEM); Student’s *t*-test. *: *p* < 0.05 and **: *p* < 0.01, and ****: *p* < 0.0001 between Sham Ovx controls and Sham non-Ovx controls (n = 6–8 mice/group). Two-way ANOVA followed by Fisher’s least significant difference (LSD) test. The results of the two-way ANOVA are presented for each variable and the Interaction (MHS × VAT or M x V). a: *p* < 0.05, b: *p* < 0.01, c: *p* < 0.001 and d: *p* < 0.0001 between MHS animals and their respective controls (Ctrl); e: *p* < 0.05, f: *p* < 0.01 and g: *p* < 0.001 between VAT animals and their sham-operated group.

## Abbreviations

The following abbreviations are used in this manuscript:

Abbreviation	Definition
AngII	Angiotensin II
Apln	Apelin
ATP2A2 (SERCA2)	Sarcoplasmic/Endoplasmic Reticulum Ca^2+^ ATPase 2
Bdh1	3-Hydroxybutyrate Dehydrogenase 1
BMI	Body Mass Index
BP	Blood Pressure
BW	Body Weight
CH	Cardiac Hypertrophy
Cilp	Cartilage Intermediate Layer Protein
Cpt1b	Carnitine Palmitoyltransferase 1B
Cpt2	Carnitine Palmitoyltransferase 2
CSA	Cardiomyocyte Cross-Sectional Area
Ctgf	Connective Tissue Growth Factor
Ctrl	Control
ECM	Extracellular Matrix
EDV	End-Diastolic Volume
EF	Ejection Fraction
ESD	End-Systolic Diameter
FAT/CD36	Fatty Acid Translocase (CD36)
FDR	False Discovery Rate
Gja1	Gap Junction Protein Alpha 1 (Connexin 43)
Glut1	Glucose Transporter 1
Glut4	Glucose Transporter 4
HFD	High-Fat Diet
HF	Heart Failure
HFpEF	Heart Failure with Preserved Ejection Fraction
HR	Heart Rate
iHeart	Indexed Heart Weight (Heart Weight/Tibial Length)
iLA	Indexed Left Atrial Weight
IL-6	Interleukin-6
iNOS	Inducible Nitric Oxide Synthase
IVSd	Diastolic Interventricular Septal Thickness
LA	Left Atrium/Left Atrial
LV	Left Ventricle/Left Ventricular
Lpx	Lipectomy
MCP-1	Monocyte Chemoattractant Protein-1 (CCL2)
MHS	Metabolic and Hypertensive Stress
miR	MicroRNA
miRNome	Global MicroRNA Expression Profile
Myh6	Alpha-Myosin Heavy Chain
Myh7	Beta-Myosin Heavy Chain
Nppa	Natriuretic Peptide A (ANP)
Nppb	Natriuretic Peptide B (BNP)
Ovx	Ovariectomized/Ovariectomy
Pdk4	Pyruvate Dehydrogenase Kinase 4
Ppia	Peptidylprolyl Isomerase A (Cyclophilin A)
Postn	Periostin
PWd	Diastolic Posterior Wall Thickness
qPCR	Quantitative Polymerase Chain Reaction
RAAS	Renin–Angiotensin–Aldosterone System
RNA	Ribonucleic Acid
RNA-Seq	RNA Sequencing
RR	Reverse Remodelling
RT-PCR	Reverse Transcription Polymerase Chain Reaction
RWT	Relative Wall Thickness
Ryr2	Ryanodine Receptor 2
SAT	Subcutaneous Adipose Tissue
SEM	Standard Error of the Mean
SNS	Sympathetic Nervous System
SV	Stroke Volume
Tgfb1	Transforming Growth Factor Beta 1
Thbs4	Thrombospondin 4
Ucp3	Uncoupling Protein 3
VAT	Visceral Adipose Tissue
VE	Voluntary Exercise
WAT	White Adipose Tissue
