# Supplementary material for: White Adipose Tissue Contributes to Cardiac Homeostasis and Adaptation to Cardiometabolic Stress in a Sex- and Depot-Specific Manner
Source: Biomolecules. 2026 Sep 19;16(9):1364. doi: 10.3390/biom16091364 (PMC13604053; doi:10.3390/biom16091364)

Supplemental data

White adipose tissue contributes to cardiac homeostasis and adaptation to cardiometabolic stress in a sex- and depot-specific manner.

Sara-Ève Thibodeau [1,2,3], Nicolas Le Bos, Élisabeth Walsh-Wilkinson [1,2,3], Emylie-Ann Labbé [1,2,3], Audrey Morin-Grandmont [1,2,3], Emmy Trahan [1,3], Mathias Zakrzewski [1,3], and Jacques Couet [1,2,3]

1 Département de Médecine, Faculté de Médecine, Université Laval, Québec City, QC G1V 0A6, Canada
2 Groupe de Recherche sur les Valvulopathies, Centre de Recherche de l'Institut Universitaire de Cardiologie et de Pneumologie de Québec, Université Laval, Québec City, QC G1V 4G5, Canada
3: Centre de recherche de l'Institut universitaire de cardiologie et de pneumologie de Québec, Université Laval, Québec City, QC, Canada
Correspondence: Jacques.couet@med.ulaval.ca (J.C.)

Table S1. Sequences of primers used in this study.

Symbol	Description	Forward sequence<br>Reverse sequence
Apln	Apelin	5'-ACC AGG ACT AGA AGG AAG C-3'<br>5'-CTG TCT GCG AAA TTT CCT CCT-3'
Atp2a2	ATPase Sarcoplasmic/Endoplasmic Reticulum Ca2+ Transporting 2	5'-GCC TGC AAC TCG GTC ATA AA-3'<br>5'-TGC ACC TAT CGA TGA CAC CT-3'
Bdh1	3-Hydroxybutyrate Dehydrogenase 1	5'-AGG ACA AAG GTG ATG CTG GG-3'<br>5'-CCA AAC GTT GAG ATG CCT GC-3'
Cilp	Cartilage Intermediate Layer Protein	5′-CTG AGA TGA AAG CAC GTC GG-3′<br>5′-AAG TAC TCC CCT GCT TGG TC-3′
Col1a1	Collagen Type I Alpha 1 Chain	5′-CAT TGT GTA TGC AGC TGA CTT C-3′<br>5′CGC AAA CAC TCT ACA TGT CTA GG-3′
Col3a1	Collagen Type III Alpha 1 Chain	5′-TCT CTA GAC TCA TAG GAC TGA CC-3′<br>5′ TTC TTC TCA CCC TTC TTC ATC C-3′
Cpt1b	Carnitine palmitoyltransferase 1b, muscle	5′-AGG CAC TTC TCA GCA TGG TC-3′<br>5′-ACG GAC ACA GAT AGC CCA GA-3′
Cpt2	Carnitine palmitoyltransferase 2	5′-CCA TGC ACT ACC AGG ACA GC-3′<br>5′ ACA CAA CAC TTC TGT CTT CCT GA-3′
Ctgf	Connective tissue growth factor	5′-GTT ACC AAT GAC AAT ACC TTC TGC-3′<br>5′-TTG ACA GGC TTG GCG ATT-3′
Fat/Cd36	Fatty Acid Translocase/CD36	5'-GAT CGG AAC TGT GGG CTC AT-3'<br>5′-CTT TGC CAC GTC ATC TGG G 3′
Gja1	Gap Junction Protein Alpha 1	5'-ACG GCA AGG TGA AGA TGA GA-3'<br>5'-GAG AGA CAC CAA GGA CAC CA-3'
Glut1	Glucose Transporter Type 1	5′-TCT CTG TCG GCC TCT TTG TT-3′<br>5′- CGC AGT ACA CAC CGA TGA TG-3′
Glut4	Glucose Transporter Type 4	5'-CAG TAC TCC CTG CTC TCC TG-3'<br>5'-GCT CTC TCT CCA ACT TCC GT-3'
Myh6	Myosin heavy chain, α isoform	5'- CTG GTC CTC CTT TAT GGT CAC -3'<br>5'- GAG TGC TTC GTG CCT GAT -3'
Myh7	Myosin heavy chain, b isoform	5'-CAG TAC TCC CTG CTC TCC TG-3'<br>5'-GCT CTC TCT CCA ACT TCC GT-3'
Nppa	Natriuretic Peptide A	5′-CTC CTT GGC TGT TAT CTT CGG-3′<br>5′-GGG TAG GAT TGA CAG GAT TGG-3′
Nppb	Natriuretic Peptide B	5′-AGG TGA CAC ATA TCT CAA GCT G-3′<br>5′-CTT CCT ACA ACA TCA GTG C-3′
Pdk4	Pyruvate Dehydrogenase Kinase 4	5'-TTC TTG AAG AGT TCG AAG AGC A-3'<br>5'-CAT CGC CAG AAT TAA ACC TCA C-3'
Postn	Periostin	5′-GCT TTC GAG AAA CTG CCA CG-3′<br>5′-ATG GTC TCA AAC ACG GCT CC-3′
Ppia	Cyclophilin A	5′-TTC ACC TTC CCA AAG ACC AC-3′<br>5′-CAA ACA CAA ACG GTT CCC AG-3′
Ryr2	Ryanodine receptor 2, cardiac	5′-AGG TGG CAG ATG CGT CTC TA-3′<br>5′-TTG AGG ATG TTC CAC CAG GC-3′
Thbs4	Thrombospondin 4	5′-GAT ACT GAC GGG GAT GGG AG-3′

Ucp3	Uncoupled protein 3	5′-CGT CAC TGT CTT GGT TGG TG-3′<br>5′-TGC GGA CCT CCT CAC TTT TC-3′<br>5′-CAA AAC TCA TCT GGC GGT GC-3′

Table S2. Echo data in male and female mice after visceral (VAT) or subcutaneous white adipose tissue (SAT) depot lipectomy. Echo exams as described in the Methods section. IVSd: diastolic interventricular septum; PWd: diastolic posterior wall thickness; ESD: end-systolic LV diameter; LV mass; HR: heart rate; CO: cardiac output; EDV: end-diastolic volume. Results are expressed as the mean ± standard error of the mean (SEM). P values were calculated using the Holm–Sidak post hoc test after a one-way ANOVA. a: p<0.05 vs sham group; b: p<0.01;c: p<0.001; d: p<0.0001. e: p<0.05 between lipectomy groups; f: p<0.01; g: p<0.001; and h: p<0.0001.

**Males**			
Parameters	**Sham (n=8)**	**VAT (n=8)**	**SAT(n=8)**
IVSd, mm	0.76±0.014	0.72±0.015	0.72±0.016
PWd, mm	0.74±0.013	0.77±0.013	0.80±0.015a
ESD, mm	3.96±0.048	3.77±0.068	4.0±0.070e
HR, bpm	509±8.0	519±15.1	532±12.7
CO, ml/min	16.0±0.71	14.8±0.63	18.2±0.99e
EDV, µl	55±2.3	48±2.4	57±1.7e
**Females**			
IVSd, mm	0.69±0.015	0.74±0.013a	0.64±0.016a,g
PW,d	0.71±0.019	0.76±0.017a	0.70±0.010e
ESD, mm	3.64±0.053	3.67±0.016	3.72±0.033
HR, bpm	520±11.6	532±12.7	500±13.0
CO, ml/min	15.7±0.63	13.2±0.65	18.2±0.99
EDV, µl	48±1.1	42±1.2b	44±1.4a

Table S3. Diastolic echo data in male and female mice after visceral (VAT) or subcutaneous white adipose tissue (SAT) depot lipectomy. Controls (C). Vehicle (Veh). Echo exams as described in the Methods section. A-wave velocity, E′ wave velocity, and A′ wave velocity. Results are expressed as the mean ± standard error of the mean (SEM). P values were calculated using the Holm–Sidak post hoc test after a one-way ANOVA. a: p<0.05 vs sham group; b: p<0.01; c: p<0.00; d: p<0.0001. e: p<0.05 between lipectomy groups; f: p<0.01; g: p<0.001; h: p<0.0001.

**Males**			
Parameters	**Sham (n=8)**	**VAT (n=8)**	**SAT(n=8)**
A, mm/s	408±19.7	339±6.5b	328±13.6b
A′, mm/s	18.9±0.48	16.9±1.03	15.2±0.54b
E/A	1.5±0.04	1.6±0.05	1.7±0.09
**Females**			
A, mm/s	361±10.8	314±19.6	335±9.9
A′, mm/s	18.5±0.78	15.3±0.41b	15.0±0.47b

E/A	1.6±0.04	1.7±0.07	1.8±0.03b

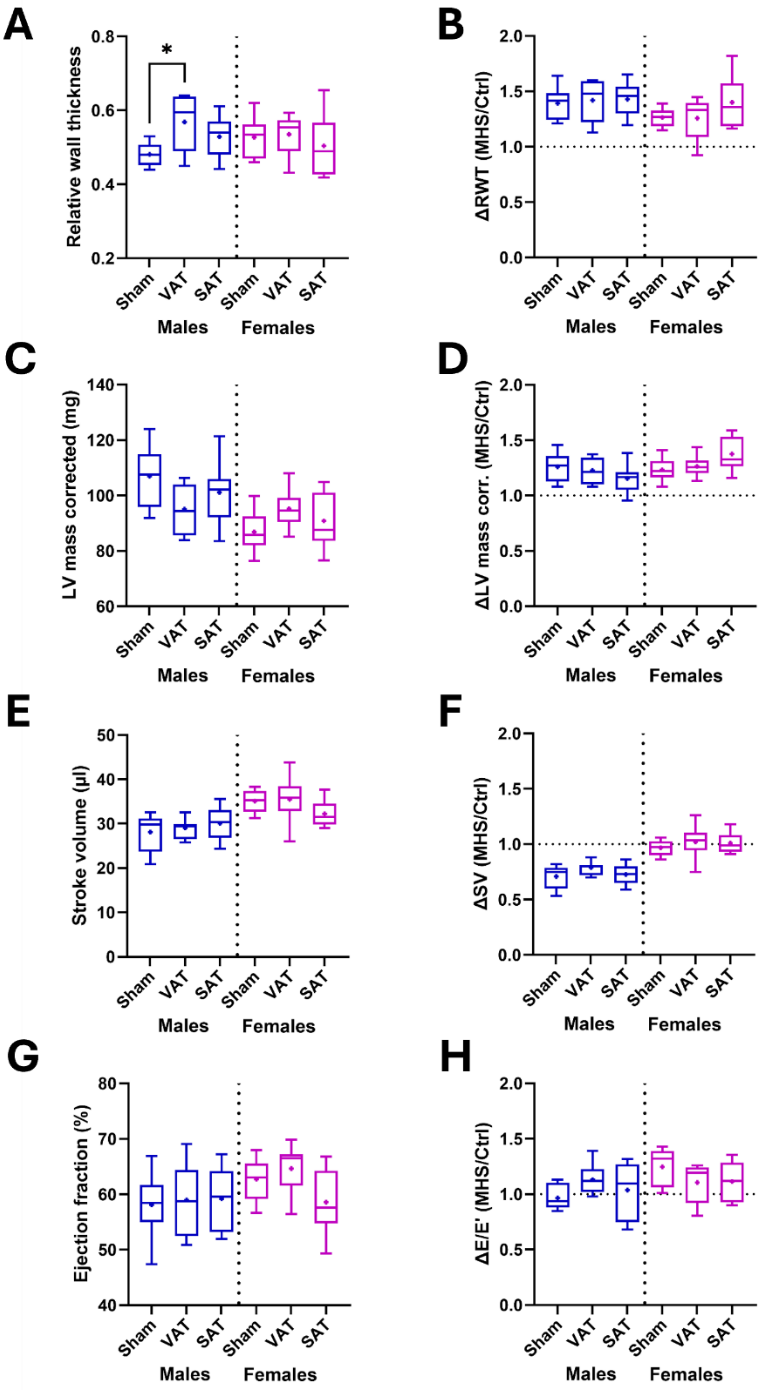

Figure S1. VAT or SAT lipectomy has little effect on echocardiography parameters in MHS males and females compared to controls. A. RWT left and right; B. DRWT; C. corrected LV mass; D. DLV mass; E. stroke volume (SV); F. DSV; G. LV ejection fraction; H. DE/E′ ratio. Males: blue and left. Females: fuchsia and right. Results are expressed as mean ± standard error of the mean (SEM); one-way ANOVA followed

by the Holm–Sidak post-test. *: p<0.05, **: p<0.01, and ***: p<0.00 between indicated groups (n=7-8 mice/group).

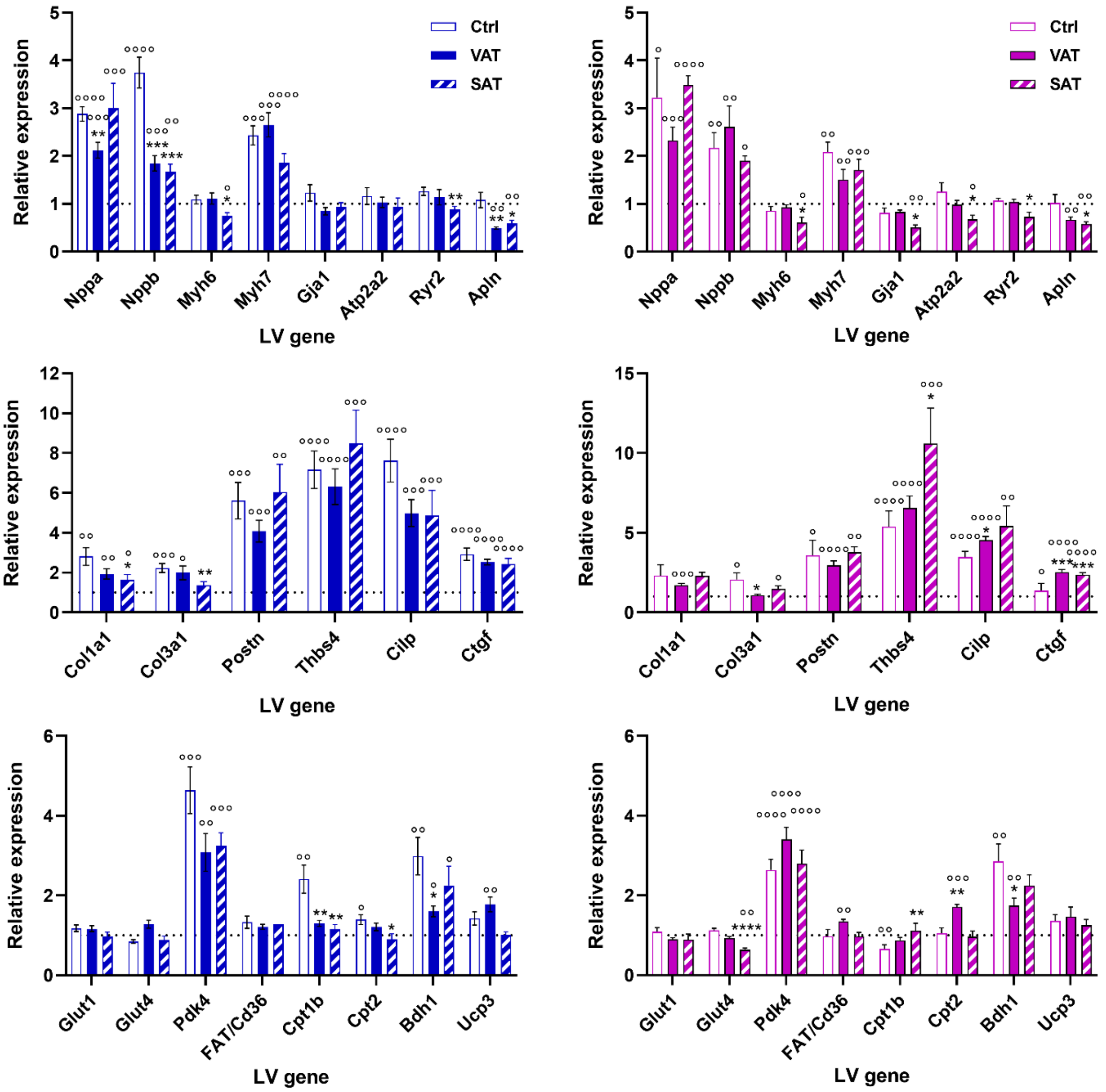

Figure S2. VAT or SAT lipectomy result in the modulation of LV genes in MHS male and female mice. A. Modulation of LV genes associated with cardiomyocyte physiology. B. LV genes associated with extracellular matrix remodelling. C. Genes implicated in myocardial energy metabolism. The dotted line in each graph represents the relative expression of the indicated gene in control mice. Results are expressed as mean ± standard error of the mean (SEM); one-way ANOVA followed by the Holm–Sidak post-test. *: p<0.05, **: p<0.01, and ***: p<0.001 between VAT or SAT groups and Sham (n=7-8 mice/group). Student's T-test. °: p<0.05, °°°: p<0.001 and °°°°: p<0.0001 between VAT or SAT animals and control (non-MHS) mice.

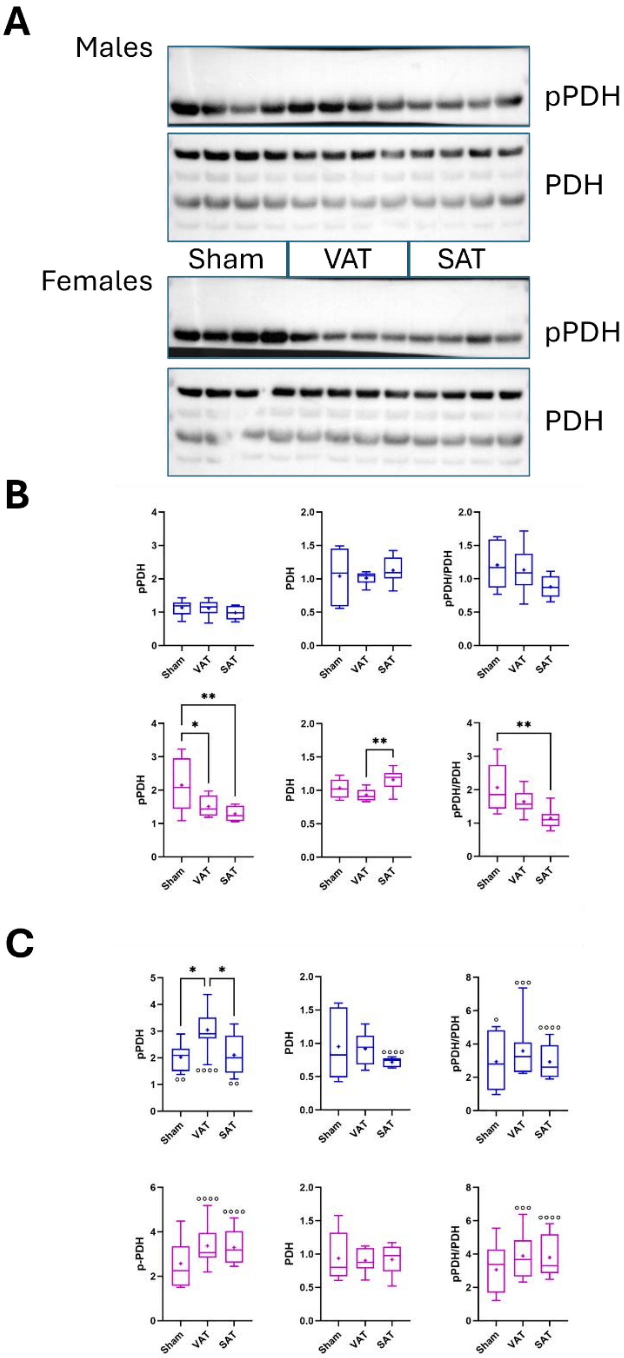

Figure S3. Impact of lipectomy on phosphorylation levels of the LV pyruvate dehydrogenase (PDH). A. Representative images of immunoblots of sham and Lpx male and female mice of phospho-PDH (pPDH) and PDH contents. B. Protein contents in control animals. C. Protein contents in MHS mice. Data are presented as mean ± SEM (n = 6–8 per group). One-way ANOVA followed by the Holm-Sidak post-test. *: p<0.05, **: p<0.01, and ***: p<0.001 between indicated groups. °: p<0.05, °°: p<0.01, °°°: p<0.001 and °°°°: p<0.0001 between indicated MHS and corresponding control group (n=7-8 mice/group).

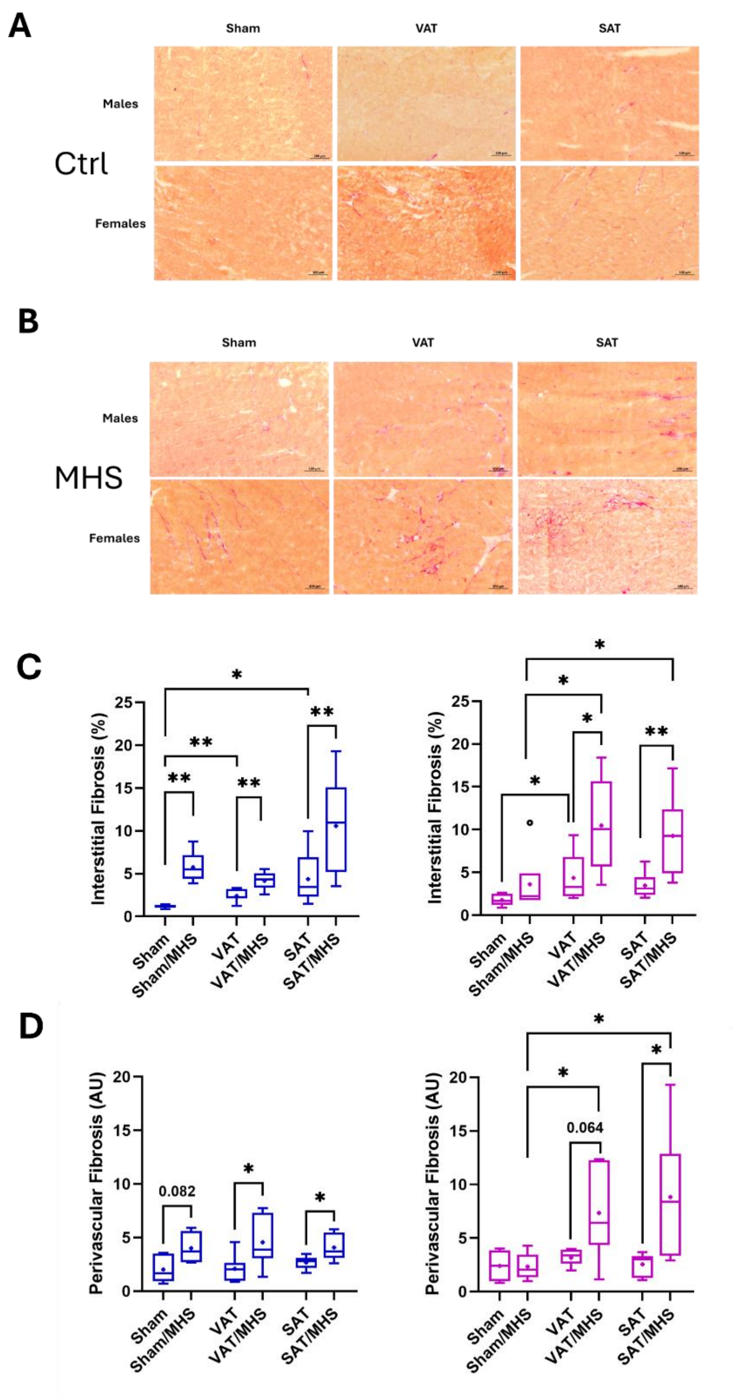

Figure S4. Lipiectomy is not associated with different levels of myocardial interstitial fibrosis in MHS mice. A. Representative images of picrosirius red staining of control male and female heart sections. B. Representative images of picrosirius red staining of MHS male and female heart sections. Interstitial fibrosis levels in control (left) and MHS (right) animals. Up: Males and females (bottom). Females: (D) Myocardial fibrosis (picrosirius red staining). Data are presented as mean ± SEM (n = 7–8 per group). One-way ANOVA followed by the Holm–Sidak post-test. *: p<0.05, **: p<0.01, and ***: p<0.001 between indicated groups (left graphs) and MHS vs. control groups (right) (n=5-7 mice/group).

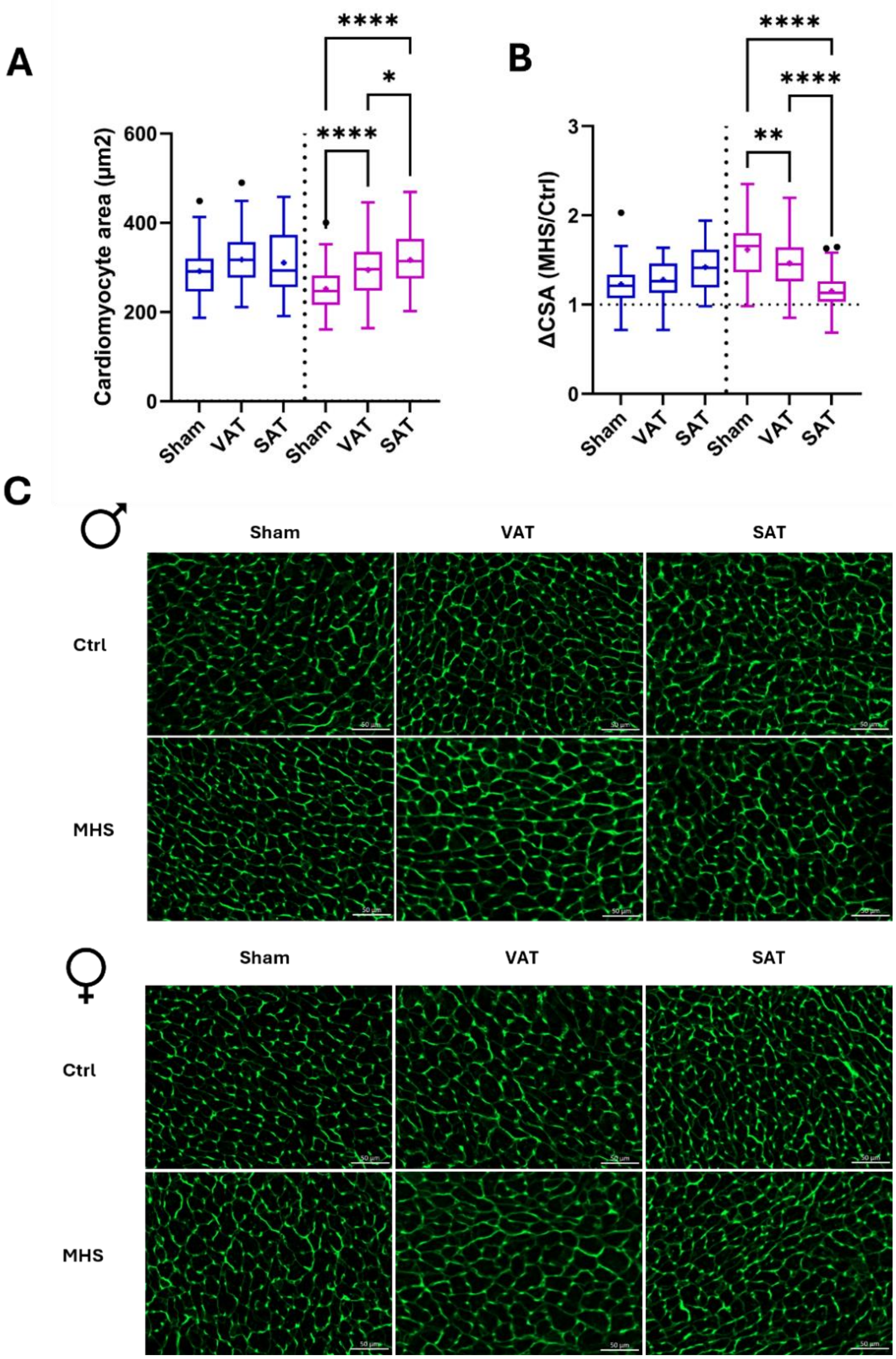

Figure S5. VAT or SAT lipectomy is associated with increased cardiomyocyte cross-sectional area in females, but not males. A. Cardiomyocyte cross-sectional area (CSA), B. DCSA. C. Representative images of WGA-FITC staining from LV sections of various indicated groups (Males). D. Females. Bar: 50μm. Data are presented as mean ± SEM (n = 7–8 per group). One-way ANOVA followed by the Holm–Sidak post-test. *: p<0.05, **: p<0.01, ***: p<0.001 and, ****: p<0.0001 between indicated groups (n=6-8 mice/group).

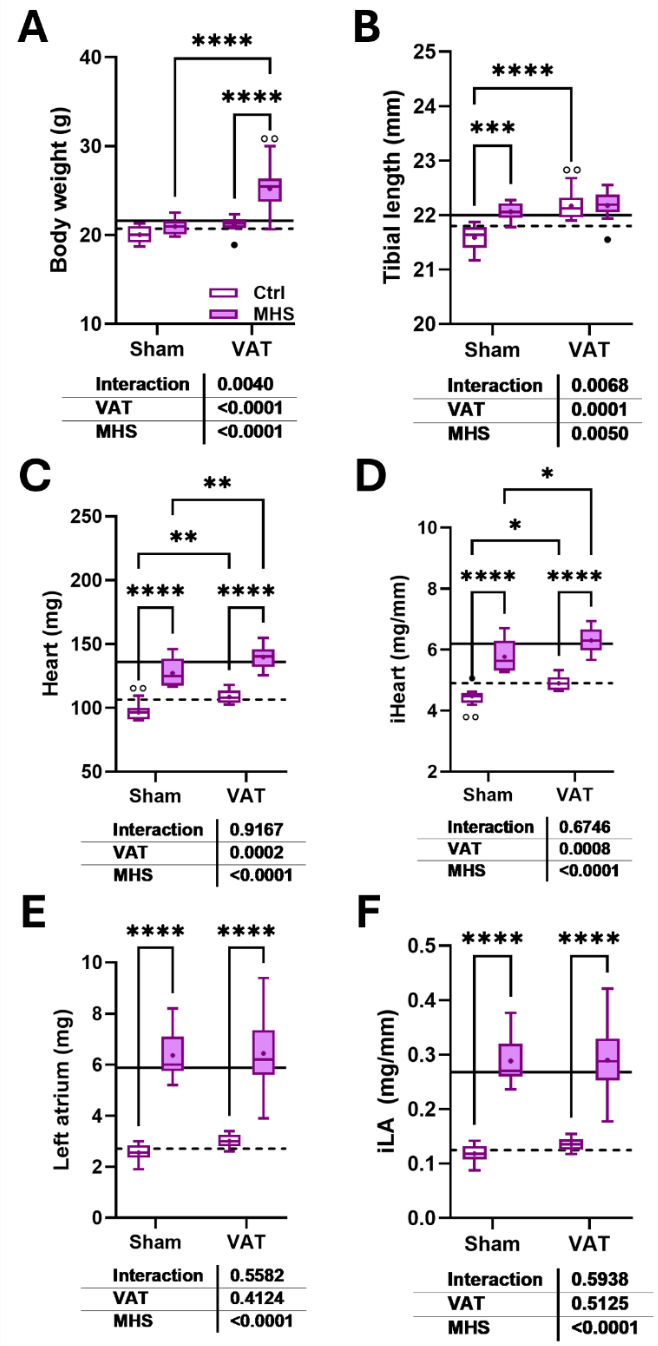

Figure S6. Removal of ovarian hormones is not associated with major differences in the mouse body and cardiac morphology in females with VAT lipectomy. A. Body weight. B. Tibial length. C. Heart weight. D. Indexed heart weight (iHeart). E. Left atrial weight and F. Indexed LA weight (iLA). The dotted line on the graphs represents the mean of the indicated parameter in control non-Ovx females, and the solid line in MHS mice. Results are expressed as mean ± standard error of the mean (SEM); two-way ANOVA followed by Fisher's least significant difference (LSD) test. The two-way ANOVA results are presented below the graph for each variable and the interaction (MHS × VAT). *: $p<0.05$, **: $p<0.01$, ***: $p<0.001$ and ****: $p<0.0001$ between indicated groups (n=7-8 mice/group). °°: $p<0.01$, between indicated MHS and corresponding control group (n=7-8 mice/group).

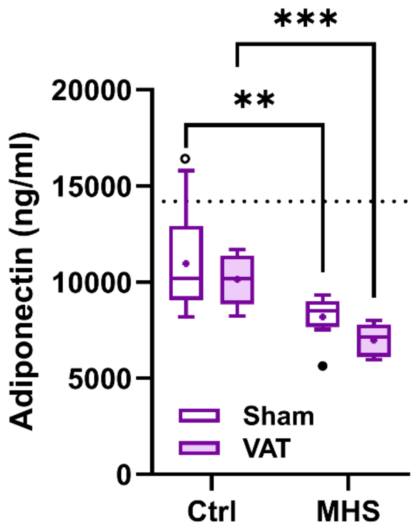

Interaction	0.7526
MHS	<0.0001
VAT	0.0811

Figure S7. VAT lipectomy modulates adiponectin levels in the circulation of ovariectomized (Ovx) mice. The dotted line represents levels in non-Ovx control females. Results are expressed as mean ± standard error of the mean (SEM); two-way ANOVA followed by Fisher's least significant difference (LSD) test. The two-way ANOVA results are presented below the graph for each variable and the Interaction (MHS × VAT). **: $p<0.01$, and ***: $p<0.001$ between indicated groups (n=7-8 mice/group). Student's T-test. °: $p<0.05$, between Ovx animals and non-OVX control mice.

Tables S4-S6. VAT or SAT lipectomy modulation of LV genes in OVX mice. A. Modulation of LV genes associated with cardiomyocyte physiology. B. LV genes associated with extracellular matrix remodelling; C. Genes implicated in myocardial energy metabolism. The dotted line in each graph represents the relative expression of the indicated gene in control mice. Results are expressed as the mean ± standard error of the mean (SEM); Student's t-test. *: p<0.05 and **: p<0.01, and ****: p<0.0001 between Sham Ovx controls and Sham non-Ovx controls (n=6-8 mice/group). Two-way ANOVA followed by Fisher's least significant difference (LSD) test. The results of the two-way ANOVA are presented for each variable and the Interaction (MHS × VAT or M x V). a: p<0.05, b: p<0.01, c: p<0.001 and d: p<0.0001 between MHS animals and their respective controls (Ctrl); e: p<0.05, f: p<0.01 and g: p<0.001 between VAT animals and their sham-operated group.

Table S4

Gene	Sham		VAT		MHS	VAT	M x V
	Ctrl	MHS	Ctrl	MHS			
Nppa	1.2±0.12	4.2±0.16d	1.7±0.15	3.0±0.58b,e	<0.0001	0.27	0.016
Nppb	0.7±0.11	1.9±0.31d	0.8±0.06	1.0±0.15f	0.002	0.023	0.0071
Myh6	0.6±0.05**	0.4±0.02b	0.4±0.03h	0.3±0.03f	0.006	<0.0001	0.23
Mhy7	0.7±0.08	2.6±0.49d	0.8±0.05	1.8±0.27b	0.0005	0.51	0.39
Gja1	1.1±0.17	0.9±0.18	0.9±0.08	0.6±0.04	0.072	0.064	0.98
Serca2	1.3±0.07*	0.8±0.07d	0.7±0.08h	0.6±0.05e	0.004	<0.0001	0.015
Ryr2	0.8±0.09	0.5±0.04b	0.6±0.03f	0.5±0.04	0.013	0.025	0.029
Apln	1.4±0.07*	1.1±0.9b	0.8±0.08h	0.9±0.04e	0.13	<0.0001	0.019

Table S5

Gene	Sham		VAT		MHS	VAT	M x V
	Ctrl	MHS	Ctrl	MHS			
Col1a1	1.2±0.24	2.0±0.49	0.4±0.03e	0.7±0.05f	0.064	0.0060	0.39
Col3a1	0.5±0.12*	1.0±0.21b	0.6±0.06	1.0±0.06	0.0013	0.73	0.42
Postn	1.0±0.19	3.2±0.66d	0.9±0.06	2.1±0.26a,e	<0.0001	0.065	0.10
Thbs4	1.6±0.16**	6.9±0.1.8b	1.5±0.15	5.8±1.3a	0.0020	0.61	0.65
Cilp	1.2±0.22	5.0±1.0d	0.7±0.06	3.1±0.18e	<0.0001	0.043	0.20
Ctgf	0.5±0.03****	1.4±0.14d	0.5±0.03	0.9±0.09c	<0.0001	0.004	0.010

Table S6

Gene	Sham		VAT		MHS	VAT	M x V
	Ctrl	MHS	Ctrl	MHS			
Glut1	0.6±0.14	0.5±0.08	0.5±0.05	0.5±0.05	0.46	0.63	0.23
Glut4	1.3±0.13*	0.9±0.04b	1.1±0.10	0.9±0.05	0.0018	0.10	0.31
Pdk4	0.5±0.09**	3.0±0.5d	0.9±0.06	2.1±0.26f	<0.0001	0.39	0.033
Cpt1a	0.5±0.04*	0.5±0.04	0.4±0.03	0.5±0.04	0.26	0.070	0.88
Cpt2	1.2±0.08	2.0±0.10c	1.2±0.20	1.2±0.10g	0.0082	0.0033	0.0030
Bdh1	1.2±0.13	1.6±0.21	1.1±0.06	1.5±0.07a	0.0069	0.31	0.89
Ucp3	0.7±0.15	1.6±0.30b	1.2±0.14	1.2±0.19	0.036	0.86	0.019

PDH (25-100 kDa)

Males

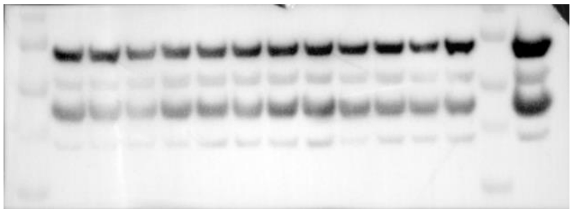
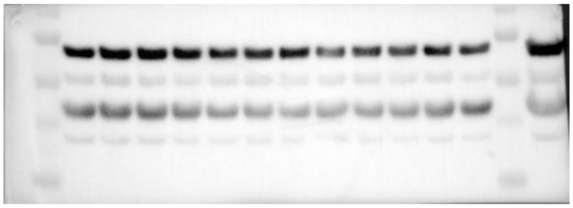

Females

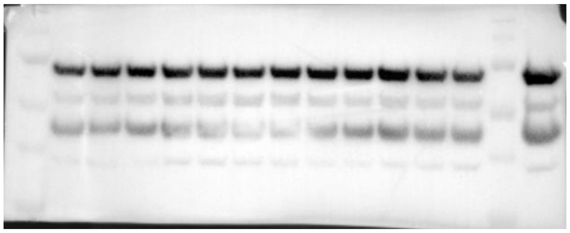
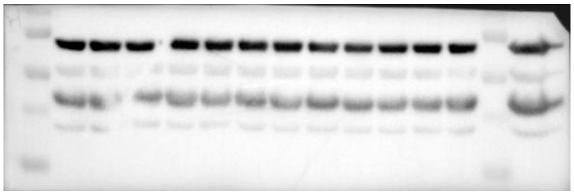

pPDH (43 kDa)

Males

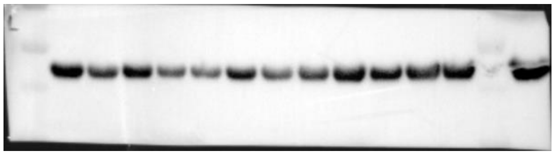
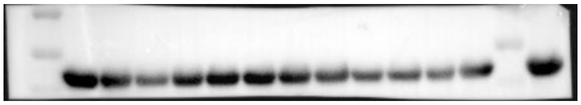

Females

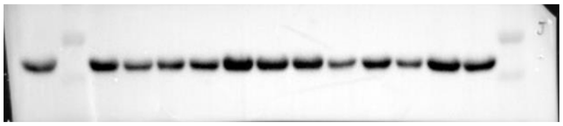
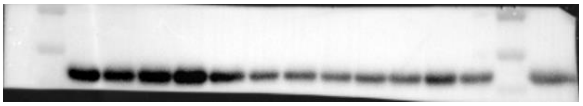

Uncropped Western blots used for results illustrated in Figure S3.

PDH

Males

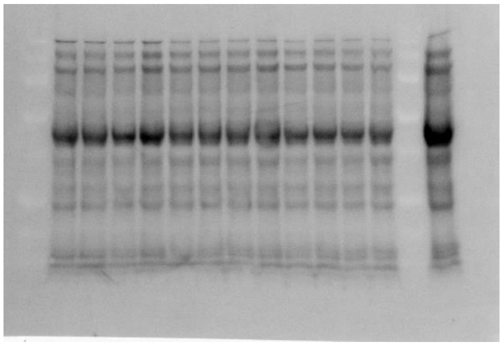
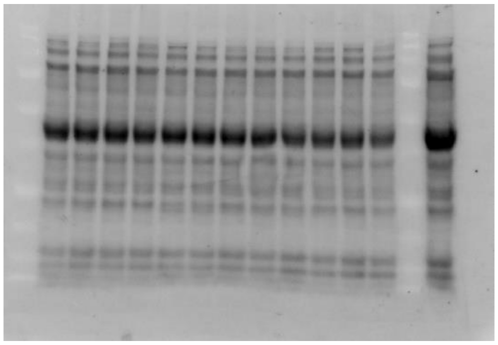

Females

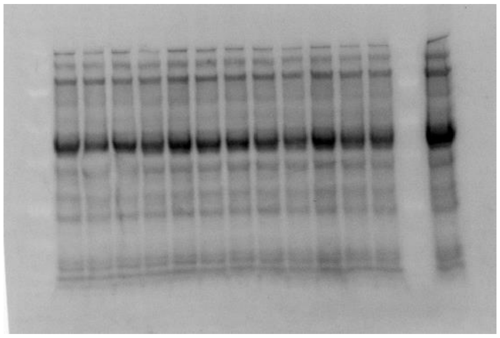
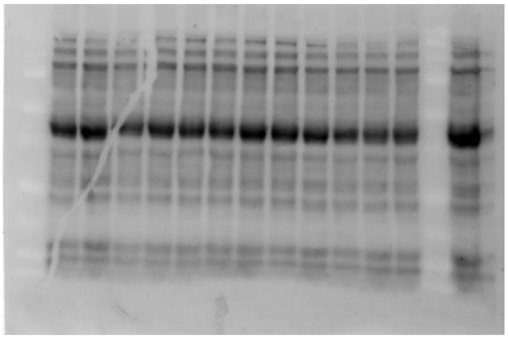

p-PDH

Males

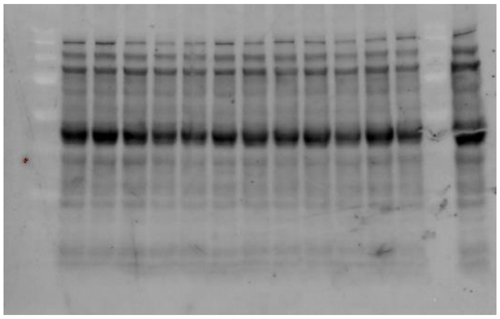
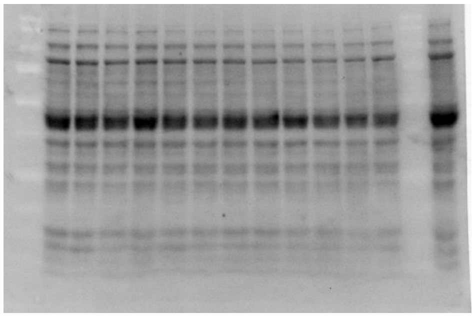

Females

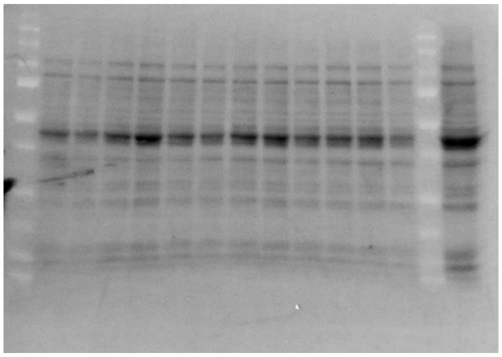
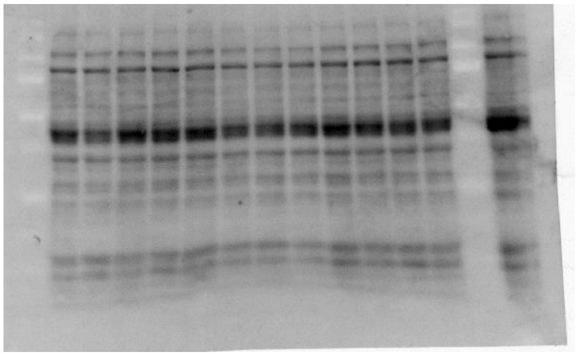

Supplement: Supplementary file 1 [file biomolecules-16-01364-s001.zip › biomolecules-4543511-supplementary.pdf]
